# Sulfur Bridge Geometry Boosts Selective Fe^IV^═O Generation for Efficient Fenton‐Like Reactions

**DOI:** 10.1002/advs.202500313

**Published:** 2025-03-05

**Authors:** Xunheng Jiang, Zhongyuan Guo, Jiang Xu, Zhiyu Pan, Chen Miao, Yue Chen, Hao Li, Hiroshi Oji, Yitao Cui, Graeme Henkelman, Xinhua Xu, Lizhong Zhu, Daohui Lin

**Affiliations:** ^1^ College of Environmental and Resource Sciences Zhejiang University Hangzhou 310058 China; ^2^ Zhejiang Provincial Key Laboratory of Organic Pollution Process and Control Zhejiang University Hangzhou 310058 China; ^3^ Advanced Institute for Materials Research (WPI‐AIMR) Tohoku University Sendai 980‐8576 Japan; ^4^ Synchrotron Radiation Research Center Nagoya University Nagoya 464‐8601 Japan; ^5^ Institute of Advanced Science Facilities Shenzhen 518052 China; ^6^ Department of Chemistry and the Oden Institute for Computational Engineering and Sciences University of Texas at Austin Austin TX 78712 USA

**Keywords:** adjacent Fe atoms, environment nanotechnology, High‐valent iron–oxo species, sulfur bridge, sustainable water treatment

## Abstract

High‐valent iron–oxo species (Fe^IV^═O) is a fascinating enzymatic agent with excellent anti‐interference abilities in various oxidation processes. However, selective and high‐yield production of Fe^IV^═O remains challenging. Herein, Fe diatomic pairs are rationally fabricated with an assisted S bridge to tune their neighbor distances and increase their loading to 11.8 wt.%. This geometry regulated the *d*‐band center of Fe atoms, favoring their bonding with the terminal and hydroxyl O sites of peroxymonosulfate (PMS) via heterolytic cleavage of O─O, improving the PMS utilization (70%), and selective generation of Fe^IV^═O (>90%) at a high yield (63% of PMS) offers competitive performance against state‐of‐the‐art catalysts. These continuous reactions in a fabricated device and technol‐economic assessment further verified the catalyst with impressive long‐term activity and scale‐up potential for sustainable water treatment. Altogether, this heteroatom‐bridge strategy of diatomic pairs constitutes a promising platform for selective and efficient synthesis of high‐valent metal–oxo species.

## Graphical abstract

The sulfur bridge regulated the geometry and electronic effects of Fe diatomic pairs for selective and efficient generation of Fe^IV^═O, opening an avenue to tune the microenvironment of active atomically dispersed sites and increase their loading amounts.

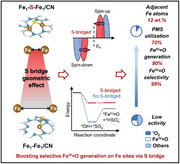

## 1 Introduction

Water security is at risk in today's world, where billions of people face water scarcity and the situation will worsen in developing regions.^[^ ^1^ ^]^ There is a pressing need to efficiently and affordably remove traditional and emerging contaminants from water for sustainable clean water supplies.^[^ ^2^ ^]^ High‐valent metal–oxo species (M^n+2^═O) which mimic the enzymes selected by nature have captivated scientists and engineers in water purification and other fields (e.g., alkane hydroxylation^[^ ^3^ ^]^ and alkene epoxidation^[^ ^4^ ^]^) via selective bond activations. M^n+2^═O provides a versatile pathway (e.g., one/two‐electron transfer,^[^ ^5^ ^]^ electrophilic addition,^[^ ^6^ ^]^ and O‐atom transfer^[^ ^7^ ^]^) and a longer lifetime than radicals in advanced oxidation processes.^[^ ^8^, ^9^ ^]^ Many efforts have been devoted to realizing the generation of M^n+2^═O via peroxymonosulfate (PMS) activation by heterogeneous catalysts.^[^ ^10^, ^11^ ^]^ However, the poor selectivity of M^n+2^═O generation restricts its application, which requires the cleavage of both O─O and O─H bonds with varied dissociation energies and puts forward higher demands on the rational design of catalysts toward PMS activation into M^n+2^═O.

Single‐atom catalysts (SACs) with tunable geometric and electronic structures can mimic natural enzymes with an efficient and selective generation of M^n+2^═O.^[^ ^9^, ^12^, ^13^ ^]^ As an element of nature's choice, Fe‐based SACs have attracted increasing interest in PMS activation.^[^ ^14^, ^15^ ^]^ Different adsorption configurations and affinities of Fe SACs toward the terminal, bridged, and hydroxyl O sites (denoted as O_ter_, O_bri_, and O_hyd_, respectively) often result in various reactive species.^[^ ^5^, ^14^, ^16^ ^]^ Highly uniform Fe─N_4_ active sites were reported to bond with O_ter_ to promote O─H bond cleavage, forming PMS radical (SO_5_^•−^) that could rapidly and selectively generate singlet oxygen (^1^O_2_) via disproportionation.^[^ ^17^, ^18^, ^19^ ^]^ In contrast, cytochromes inspired Fe─N_5_ active sites with lower *d*‐orbital occupancy tended to activate O_bri_ and produce Fe^IV/V^═O via the heterolysis/homolysis of O─O bond cleavage,^[^ ^5^ ^]^ despite the Fe loading being relatively low (< 1.5 wt.%). Meanwhile, the electron‐transfer process has also been reported in similar Fe SACs/PMS systems.^[^ ^20^ ^]^ These findings indicate that the activation pathway of PMS depends on the coordination environment of Fe sites. Thus, to achieve a highly selective generation of Fe^IV^═O, further investigation on how these Fe sites interact with O sites is still needed. A single Fe atom site does not facilitate multiple energetically favorable steps of proton‐electron coupling transfer during successive cleavage of O─O and O─H bonds. Recently, adjacent Fe sites with a specific Fe_1_–Fe_1_ distance (4–5 Å) and enhanced electron transfer have presented a selective generation of Fe^IV^═O via the O_hyd_ activation to form an inter‐complex, where the Fe loading, PMS utilization, Fe^IV^═O selectivity, and Fe^IV^═O yield was 3.1 wt.%, 36%, 92%, and 1.24 mmol per mol PMS, respectively.^[^ ^14^ ^]^ However, the distance between Fe_1_–Fe_1_ atoms supported on the same carriers is hard to control due to their high surface free energy and strong metal‐metal interaction.^[^ ^21^, ^22^ ^]^ Moreover, these adjacent Fe atoms can easily form clusters with high electron density, especially at high metal loading,^[^ ^23^, ^24^ ^]^ leading to nonselective production of radicals via O─O bond cleavage and decreasing the selectivity of Fe^IV^═O generation. Consequently, developing adjacent Fe sites with a suitable distance, a lowered charge distribution in active sites, and high loading are challenging but crucial for efficient and selective Fe^IV^═O generation.

Bridging different atomic configurations with an extra atom provides a strategy to eliminate the dilemma of adjacent metal sites.^[^ ^25^, ^26^, ^27^ ^]^ A bridged atom's geometric and electronic effects on two adjacent metal sites could offer an inhibited tendency to form the clusters at high loadings, and their microenvironment could be modified to work cooperatively to adsorb and activate target molecules.^[^ ^28^, ^29^, ^30^ ^]^ Sulfur (S) atom can effectively anchor metal sites and retarded their migration, coalescence, and Ostwald processes.^[^ ^31^, ^32^ ^]^ Moreover, S often acts as an electron‐deficient mediator when it attaches to electron‐rich metal atoms (e.g., Fe), which could regulate the charge occupancy at *d*‐orbitals and change the adsorption of reactants/intermediates at their O sites.^[^ ^33^, ^34^ ^]^ These findings suggest that bridging adjacent Fe atoms with S holds promise toward elaborately tuning their arrangement and selective generation of Fe^IV^═O via PMS activation. The bridged S content would also have an impact on the loading of Fe‐single‐atom sites and the yield of Fe^IV^═O. Therefore, constructing high‐loading S‐bridged Fe sites with a suitable distance and *d*‐orbital electronic structure for preferential cleavages of both O─O and O─H bonds can be a feasible solution for the selective and high‐yield generation of Fe^IV^═O.

Strong metal‐support interaction and high melting point can contribute to preparing high‐loading (>3 wt.%) and controllable coordination configuration (M–N_X_) of metal SACs via supermolecule self‐assembly.^[^ ^35^ ^]^ Herein, we reported a “ligand‐preselected” wet‐chemistry strategy to introduce an S bridge between adjacent Fe atoms on a graphitic carbon nitride (g–C_3_N_4_) support with a high Fe loading (i.e., 11.8 wt.%). Experimental and theoretical results together demonstrated that the bridging S, more than a pivot to disperse and stabilize adjacent Fe atoms finely, can also regulate the geometric atomic distance and *d*‐orbital electronic structure of Fe active sites. Synergistic interactions of neighboring Fe single atoms via bridging S present an outstanding reactivity for contaminants degradation, high PMS utilization, and selective generation of Fe^IV^═O with a high yield. This study identifies a fundamental mechanism of selective and high‐yield Fe^IV^═O generation via PMS activation and highlights a bridging means between adjacent Fe atoms to improve this.

## 2 Results and Discussion

### 2.1 Distribution and Distance of S‐Bridged Fe Atoms

**Figure 1:**
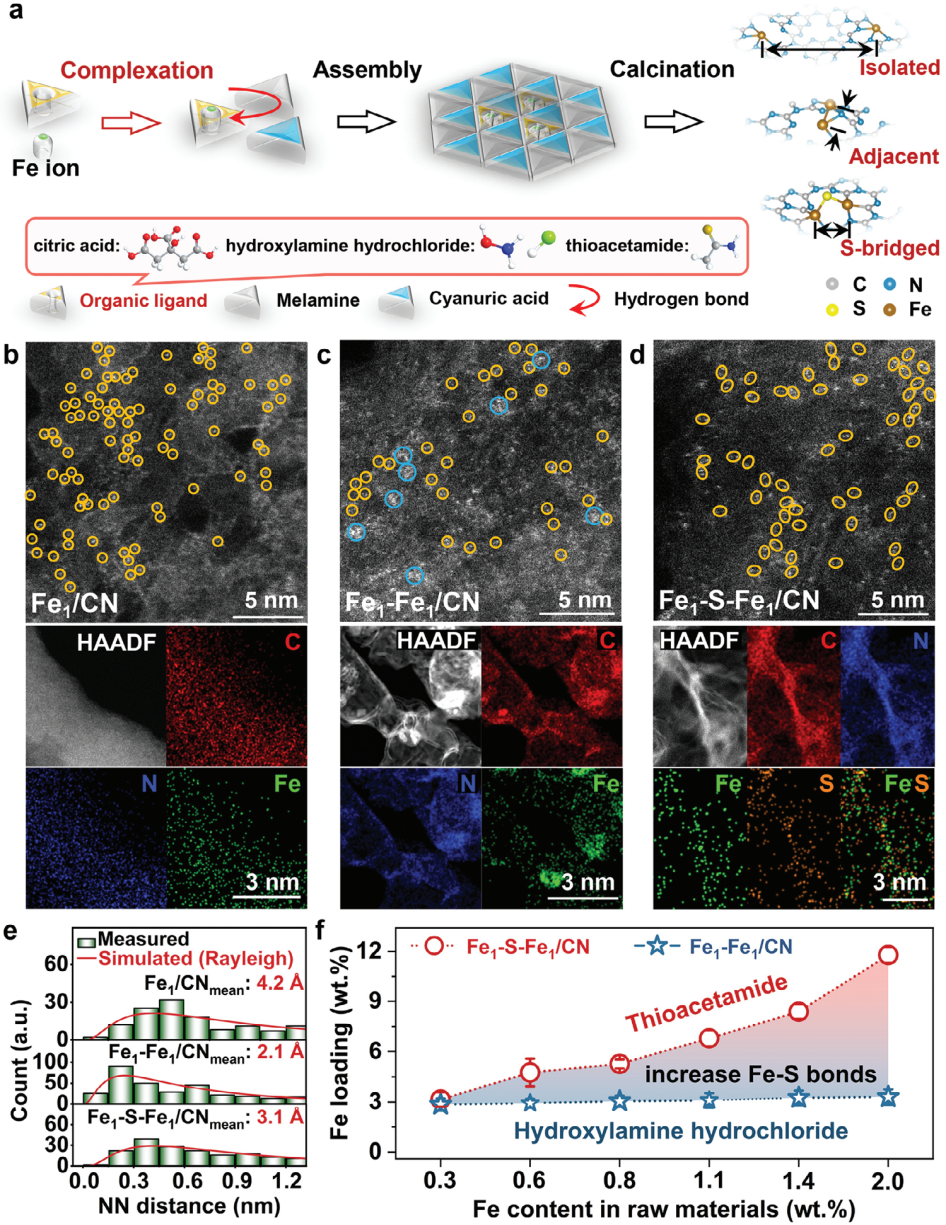
Geometry characterizations of single Fe atoms, adjacent Fe atoms, and S‐bridged Fe atoms. a) Illustration of the distance between Fe atoms in varied catalysts. AC‐HAADF‐STEM images and elemental maps of b) Fe_1_/CN, c) Fe_1_–Fe_1_/CN, and d) Fe_1_–S–Fe_1_/CN. e) Distributions of nearest neighbor distances between Fe atoms in different catalysts. f) Comparison of Fe loading between Fe_1_–Fe_1_/CN and Fe_1_–S–Fe_1_/CN catalysts under different Fe contents in raw materials.

A ligand‐assisted supermolecular self‐assembly process was applied to bridge isolated Fe atoms with S, suppressing their migration and coalescence as well as controlling the distance of two adjacent Fe atoms in g–C_3_N_4_ (**Figure** **1**a). The organic ligands play a pre‐anchoring role in this process. Specifically, citric acid (CTA) is a weak acid, and its mild complexing reaction with ferric ions was conducive to the formation of homogeneous Fe SAC.^[^ ^13^ ^]^ Hydroxylamine hydrochloride (HH) is relatively more acidic, and both HCl and NH_2_OH in its structure can competitively complex with ferrous ions, favoring the formation of adjacent Fe atoms. Compared to the N─H (σ bond), the C═S (π bond) in the structure of weakly basic thioacetamide (TCA) is more favorable to coordinate with ferrous ions, promoting the formation of Fe─S bond without Fe─Fe bond and subsequent S bridging between adjacent Fe atoms. The geometries and configurations of atomically dispersed Fe sites (Fe_1_/CN), adjacent Fe sites without S (Fe_1_–Fe_1_/CN), and S‐bridged adjacent Fe sites (Fe_1_–S–Fe_1_/CN) were examined using aberration‐corrected high‐angle annular dark‐field scanning transmission electron microscopy (AC‐HAADF‐STEM). The collected image of Fe_1_/CN showed many individual Fe atoms (marked with yellow circles) (Figure 1b), and the corresponding elemental maps indicated their atomic distribution across the layered g–C_3_N_4_ supports. Although adjacent Fe atoms were observed in the Fe_1_–Fe_1_/CN catalyst (Figure 1c), not all of them were diatomic pairs (marked with yellow circles) and some Fe formed clusters (marked with blue circles), which is likely due to the high‐loading. In contrast, diatomic Fe pairs (like dimers marked with yellow circles) with few clusters were realized by the S bridge (Figure 1d), where Fe and S atoms were homogeneously dispersed in the Fe_1_–S–Fe_1_/CN, implying that the S bridge can successfully inhibit the potential agglomeration of adjacent Fe atoms.

Bridging S could effectively regulate the distance between neighboring Fe atoms. A statistical approach comparing the measured and theoretical (Rayleigh distributions) nearest neighbor (NN) distance between Fe atoms was adopted to gain more quantitative insights (Figure 1e; Text S1, Supporting Information).^[^ ^36^, ^37^ ^]^ The average NN distance of Fe_1_/CN was 4.2 Å as expected in the presence of isolated Fe atoms in different heptazine rings.^[^ ^38^ ^]^ This distance was significantly shortened to 2.1 Å in Fe_1_–Fe_1_/CN owing to high surface energy and proximity of gathered Fe atoms. Interestingly, the S atoms in Fe_1_–S–Fe_1_/CN ulteriorly expanded the average NN distance to 3.1 Å, which is probably caused by two *Z*‐axis non‐planar Fe─S coordination bonds, as discussed later. In addition, changing the Fe precursor dose with the small chelating capacity of HH did not improve the Fe loading of Fe_1_–Fe_1_/CNs, but significantly increased the Fe loading of Fe_1_–S–Fe_1_/CNs from 3.2 wt.% to 11.8 wt.% in the presence of TCA with relatively large chelation capacity (Figure 1f; Table S1, Supporting Information). These results further indicated that the TCA could increase the loading of adjacent Fe atoms with inhibited cluster formation. The high loading of Fe atoms in Fe SACs would benefit the effective generation of Fe^IV^═O at a high yield, as discussed later.

### 2.2 Atomic Structure and Chemical State of S‐Bridged Fe Atoms

**Figure 2:**
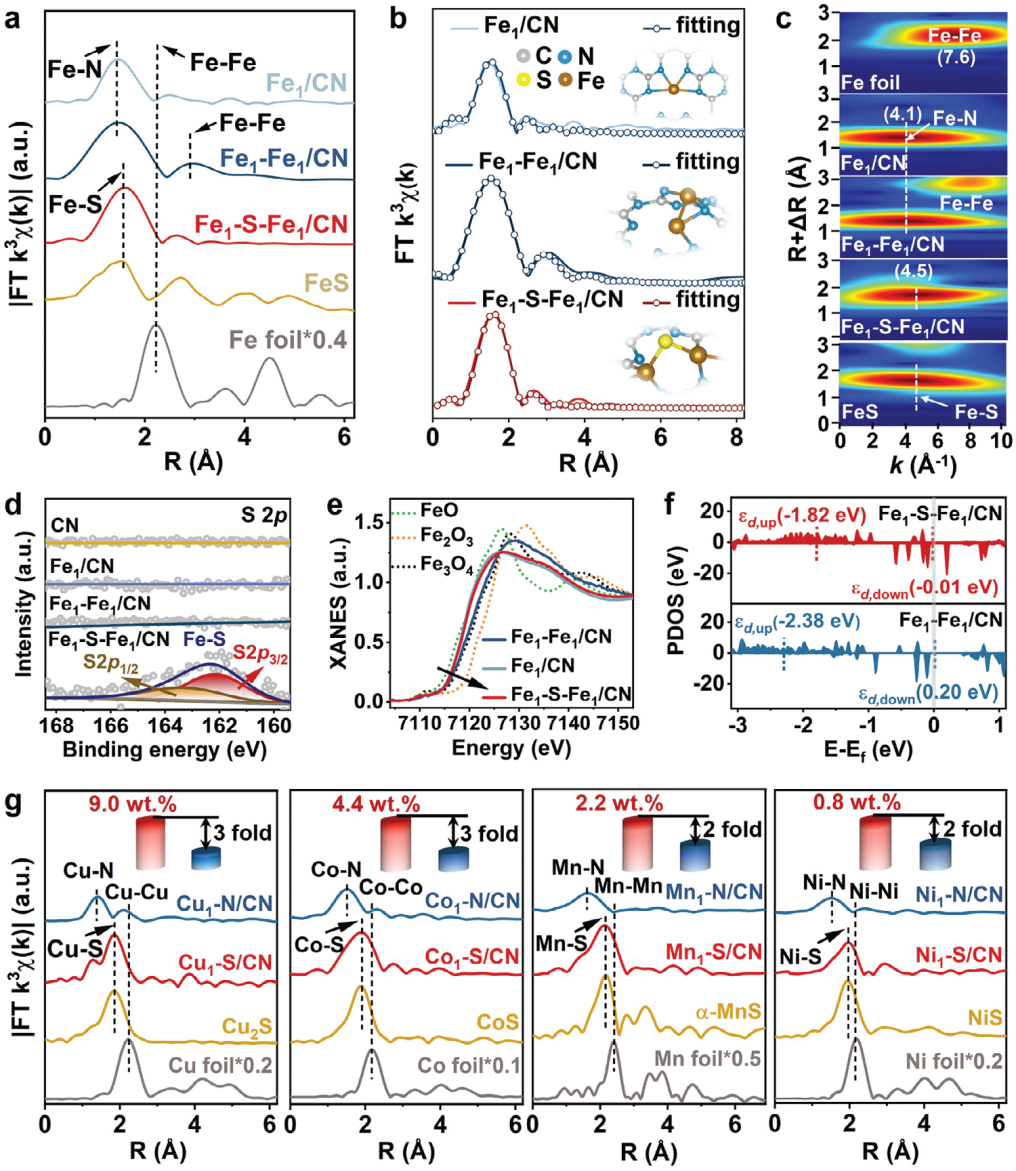
Atomic structural and chemical states analysis of as‐prepared catalysts. a) Fourier‐transformed magnitudes (FT|*k* ^3^χ(k)|) of the experimental Fe *K*‐edge EXAFS signals of catalysts and relevant references (Fe foil and FeS). b) The corresponding EXAFS fitting curves in *R*‐space, along with the schematic Fe atomic structure models. c) WT plots of catalysts and relevant references. d) S 2*p* XPS spectra of catalysts. e) Fe *K*‐edge XANES spectra of catalysts and relevant references. f) Projected density of states (PDOS) of Fe atoms in Fe_1_–Fe_1_/CN and Fe_1_–S–Fe_1_/CN. The grey‐filled line corresponds to the Fermi level (*E*_f_ = 0), and the *d*‐band center is also suggested with the dashed line. g) Fourier‐transformed EXAFS spectra of atomic metal sites with or without S coordination. Insert: Loading comparison of M_1_–S/CN and M_1_–N/CN (M = Cu, Co, Ni, or Mn).

The coordination environments of Fe atoms in the catalysts with the highest Fe loading were identified at an atomic level by the extended X‐ray absorption fine structure (EXAFS) analyses (**Figure** **2**a). The presence of Fe─N scattering path and the absence of Fe─Fe scattering path suggest that the Fe atoms were atomically dispersed in the Fe_1_/CN (8.9 wt.%), as reported in our recent work.^[^ ^13^ ^]^ While the notable Fe─N peak (1.5 Å) and an additional minor Fe─Fe peak (2.9 Å) appeared, indicating the form of adjacent Fe─Fe sites (or even a few Fe clusters as displayed in Figure 1c) along with atomic Fe─N sites in the Fe_1_–Fe_1_/CN (3.3 wt.%).^[^ ^5^, ^14^, ^39^, ^40^ ^]^ The Fourier‐transformed (FT) EXAFS of Fe_1_–S–Fe_1_/CN exhibits a prominent peak at 1.6 Å in the *R* space, belonging to the Fe─S scattering path, which agrees well with the FeS reference (Figure 2a). The fit EXAFS model shows that the Fe_1_/CN was dominated by the Fe─N_4_ environment (Figure 2b; Table S2, Supporting Information). Notably, the Fe─Fe coordination number was 0.98 in the Fe_1_–Fe_1_/CN, suggesting that most Fe was probably bound with a Fe atom. To further verify the precise Fe─S coordination number, these possible Fe─Fe or Fe─S scattering paths of EXAFS in *R*‐space were fitting (Figure S1; Table S2, Supporting information). The Fe─S coordination numbers around 0.5 without Fe─Fe (*R* factor of χ1 is abnormal) or Fe─S (total coordination numbers of χ2 is excessive) paths, which further confirm the proposed structure that two adjacent Fe atoms were anchored on one S atom in the Fe_1_–S–Fe_1_ (11.8 wt.%), as well as to C and N atoms. EXAFS wavelet transforms (WT) plot was then used to distinguish the backscattering atoms with high resolution in both *K* and *R* space (Figure 2c). Fe_1_/CN and Fe_1_–Fe_1_/CN display an intensity maximum at 4.1 Å^−1^ was attributed to the Fe─N bond. This maximum intensity shifted to 4.5 Å^−1^ in Fe_1_–S–Fe_1_/CN due to the presence of the Fe─S bond at the first coordination shell, unlike the Fe─S peak at 4.5 Å^−1^ according to two R‐values of FeS reference.

Before gaining their atomic configurations, multiple characterization methods were applied to further investigate whether the Fe and S positions were on the carrier or not. X‐ray diffraction (XRD) patterns and Fourier‐transformed infrared spectrum (FTIR) of Fe SACs display corresponding spectral features associated with the g–C_3_N_4_ carrier (Figures S2 and S3, Supporting Information), respectively, suggesting that the highly dispersed Fe species did not significantly change the main framework of carriers.^[^ ^18^, ^41^ ^]^ However, the enlarged XRD patterns captured a gradual blue shift of the (100) plane (i.e., from 12.8° to 12.5°) after the introduction of single Fe atoms, adjacent Fe atoms, and S‐bridged Fe atoms, indicating the Fe coordination with C and N atoms in the triazine ring of g–C_3_N_4_ carrier,^[^ ^42^, ^43^ ^]^ which is consistent with the EXAFS and WT analyses (Figure 2a–c). In contrast, incorporating single Fe atoms in the Fe_1_/CN did not change the indexed (002) diffraction peaks compared to that of the g‐C_3_N_4_ carrier. In contrast, the adjacent and S‐bridged Fe atoms induced obvious shifts. Since the (002) plane corresponds to the interlayer characteristic of g–C_3_N_4_,^[^ ^44^, ^45^ ^]^ this difference implies that the single Fe atoms tended to present in a single g–C_3_N_4_ layer, and some Fe and S atoms in the Fe_1_–S–Fe_1_/CN were likely apt to stay away from a single layer. The S 2*p* X‐ray photoelectron spectroscopy (XPS) further confirmed this (Figure 2d), where only Fe─S ascribed peaks (i.e., S 2*p* ^3/2^ at 162.1 eV and S 2*p* ^1/2^ at 163.2 eV)^[^ ^46^ ^]^ was observed without any S‐containing impurity peaks (i.e., C─S─C(N) or C─S─O_X_).^[^ ^31^, ^33^, ^47^ ^]^ These characterizations also indicate successfully constructing a bridging S between Fe atoms in the catalyst. Based on the above results, the detailed atomic configurations of Fe atoms with or without S were established (as shown in the insert of Figure 2b), which were adopted for the following theoretical calculations.

The bridging S‐induced electronic effects were then investigated based on the above geometry observations. S atoms can usually act as a tailor to manipulate electron delocalization and change the d‐orbital center of Fe atoms.^[^ ^33^, ^34^, ^48^ ^]^ The Fe *K*‐edge X‐ray absorption near edge structure (XANES) profiles (Figure 2e) illustrate that the positions of the absorption edge (onset of the spectra) of Fe_1_/CN, Fe_1_–Fe_1_/CN, and Fe_1_–S–Fe_1_/CN were located between FeO and Fe_2_O_3_. The Fe valence state of Fe_1_/CN (2.63), Fe_1_–Fe_1_/CN (2.96), and Fe_1_–S–Fe_1_/CN (2.68) calculated as the first derivative of XANES (Figure S4, Supporting Information),^[^ ^49^ ^]^ which further suggests that the carried positive charges of Fe were tuned by the adjacent Fe atom and bridging S.^[^ ^34^, ^48^ ^]^ Since the strong interaction between Fe atoms, the Fe_1_–Fe_1_ pairs with high electron density were formed and each Fe atom was still N‐coordination within Fe_1_–Fe_1_/CN, which promoted electron transfer and increased the valence state of Fe atoms compared to Fe_1_/CN. In contrast, the bridging S atom was more likely could transfer electrons to Fe atom than N atom, resulting in a lower valence state for Fe atoms in Fe_1_–S–Fe_1_/CN rather than Fe_1_–Fe_1_/CN. The C 1s and N 1s XPS spectra present that the introduction of individual and adjacent Fe atoms did not change the N─C═N peak but caused an upshift of the main C─N═C peak (Figure S5a,b, Supporting Information). Both the N─C═N and C─N═C peaks shifted toward higher binding energy for the Fe_1_–S–Fe_1_/CN caused by the coordination of Fe sites with C and N, consistent with the result of EXAFS spectra (Figure 2a). These bridging S‐induced electronic effects were evaluated by the projected density of states (PDOS) of the Fe atoms in catalysts (Figure 2f). Specifically, the bridging S elevates the *d*‐band center of Fe atoms from −2.38 to −1.82 eV for spin‐up electrons, and 0.20 to −0.01 eV for spin‐down electrons. This can be attributed to the bridging S atom reduced the electron localization of Fe atoms via the *d‐p* orbital coupling, bringing its *d*‐orbital distribution closer to the Fermi level, which could optimize the adsorption of PMS on the Fe_1_–S–Fe_1_/CN catalyst, as discussed later. The relative energy level of each *d*‐orbital was further investigated by projecting the DOS onto different 3*d* orbitals (Figure S6, Supporting Information), showing that the electronic interaction mainly occurred between *d*z^2^ and *d*xz in Fe_1_–Fe_1_/CN, and the PDOS of these *d*‐orbitals (i.e., *d*xz and *d*xy) was also closer to the Fermi level in the presence of bridging S. This might contribute to intermediate adsorption between Fe_1_–S–Fe_1_/CN and multiple O sites of PMS via electronic interactions in the *d*_XZ_ and *d*xy orbitals.^[^ ^50^ ^]^ The universality of S coordination with metals theoretically makes it possible to prepare a list of single‐atom catalysts with increased loading, if the geometry and electronic effects are reasonably adjusted via selective ion coordination in solution. For instance, S‐ and N‐ coordinated‐metal SACs on CN were successfully obtained by replacing FeSO_4_ with divalent metal salt soluble in water (i.e., CuSO_4_, CoSO_4_, NiSO_4_, or MnSO_4_) (Figure 2g). Analogously, this S‐coordination strategy significantly increased the atomic loading of these transition metals by 2–3 fold (up to 9 wt.%). These engineered coordinated environments of Fe atoms with regulated geometric and electronic effects allow a tunable generation of reactive species, as described below.

### 2.3 Identification and Selective Generation Mechanism of Fe^IV^═O Species

**Figure 3:**
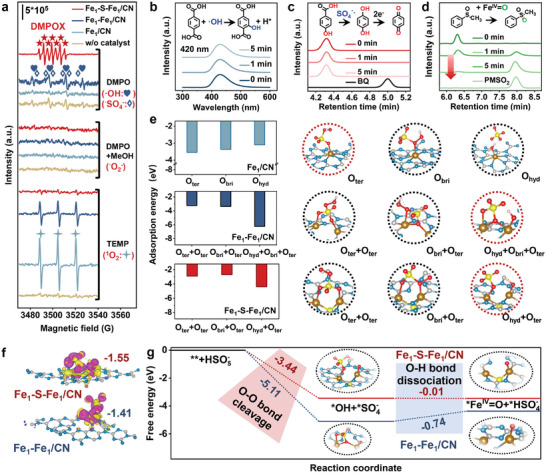
Identifications of reactive oxygen species and mechanism of selective Fe^IV^═O generation. a) EPR spectra of catalysts/PMS systems with trapping agents. DMPO = TEMP = 50 mM. b) Fluorescence emission spectra of the Fe_1_–S–Fe_1_/CN/PMS system for quantitative detection of •OH with a photoexcited wavelength of 315 nm. High‐performance liquid chromatography for quantitative detection of c) the SO_4_^•−^ and d) Fe^IV^═O. e) Calculated PMS adsorption energies onto different O sites by Fe_1_/CN, Fe_1_–Fe_1_/CN, and Fe_1_–S–Fe_1_/CN catalysts (inserts are adsorption configurations of *PMS). f) Charge density differences induced by PMS adsorption on Fe_1_–Fe_1_/CN and Fe_1_–S–Fe_1_/CN (electron accumulation and depletion were expressed by the purple and yellow iso‐surfaces, respectively. Isosurface = 0.003 *e*/Bohr^3^). g) Free energy profiles of Fe^IV^═O generation via the PMS activation by Fe_1_–Fe_1_/CN and Fe_1_–S–Fe_1_/CN catalysts.

The reactive species generated via the activation of PMS by the Fe_1_–S–Fe_1_/CN catalysts were identified by electron paramagnetic resonance (EPR) spectroscopy, applying 5,5‐dimethyl‐1‐pyrrolidine‐N‐oxide (DMPO), methanol (MeOH), and 2,2,6,6‐tetramethylproline (TEMP) as the trapping agents. While the only triplet signal of TMEP‐^1^O_2_ detected in the Fe_1_/CN/PMS system suggests the highly selective generation of ^1^O_2_, the characteristic signals of DMPO‐•OH, DMPO‐SO_4_^•−^, and TMEP‐^1^O_2_ indicate various reactive species in the Fe_1_–Fe_1_/CN/PMS system (**Figure** **3**a). For the Fe_1_–S–Fe_1_/CN/PMS system, no DMPO‐•OH, DMPO‐SO_4_^•−^, or DMPO‐O_2_^•−^ signal was detected, and the generation of ^1^O_2_ was not favored according to the limited signal of TMEP‐^1^O_2_. Rather, a heptet signal of 5,5‐dimethylpyrrolidone‐2‐(oxy) (DMPOX) with an intensity of 1:2:1:2:1:2:1 indicated the direction oxidation of DMPO via a nonradical pathway.^[^ ^51^, ^52^ ^]^ Furthermore, the signals of the radical probes, i.e., terephthalic acid (TA) for hydroxyl radical (•OH) and hydroxybenzoic acid (HBA) for sulfate radical (SO_4_^•−^),^[^ ^18^, ^53^ ^]^ were not significantly changed during the oxidation reaction, suggesting their incident formation (Figure 3b,c). Notably, the Fe^IV^═O probe phenyl methyl sulfoxide (PMSO) gradually decreased along with the increase of phenyl methyl sulfone (PMSO_2_) (Figure 3d). Likewise, the appearance of Fe(IV)‐complex at around 776 cm^−1^ in the Fe_1_–S–Fe_1_/CN/PMS system and inexistence in the Fe_1_/CN/PMS system no matter the extension of reaction times (Figure S7, Supporting Information).^[^ ^14^, ^54^ ^]^ The Raman results indicate the dominant contribution of Fe^IV^═O to the contaminant oxidation in the Fe_1_–S–Fe_1_/CN/PMS system, where oxygen transfer reactions occurred.^[^ ^7^ ^]^ These findings confirmed that the incorporation of bridging S atoms into the local coordination environment of Fe SACs favored the selective generation of Fe^IV^═O.

Theoretical computations further explored the possible pathway toward selective Fe^IV^═O production. Since the activation pathways of PMS could be changed by its O binding sites,^[^ ^5^, ^14^, ^16^ ^]^ the impacts of bridging S on adsorption configurations of PMS by three O sites (i.e., O_ter_, O_bri_, and O_hyd_) were investigated (Table S3, Supporting Information). The most favorable configuration by O_ter_‐binding on Fe_1_/CN (Figure 3e) was consistent with our previous study,^[^ ^18^ ^]^ indicating nearly 100% selectivity of ^1^O_2_ generation via the disproportion of SO_5_^•−^. The bonding configurations of O_ter_ + O_bri_ + O_hyd_ and O_ter_ + O_hyd_ atoms became more stable when PMS molecules approached adjacent Fe atoms (Figure 3f) and S‐bridged adjacent Fe atoms (Figure 3g), respectively. This preferentially binding configuration of PMS on Fe_1_–Fe_1_/CN would lead to the spontaneous decomposition of PMS into SO_4_^•−^ and •OH via the O─O bonds cleavage,^[^ ^17^, ^18^ ^]^ which agrees with our EPR results (Figure 3a) and quenching experiments, as discussed below. While both O_ter_ and O_hyd_ atoms bond to one Fe atom in the Fe_1_–S–Fe_1_/CN, one of the S‐bridged adjacent Fe atoms only bonds to one O atom via the heterolytic cleavage of O─O. The latter configuration would facilitate the following O─H cleavage to produce Fe^IV^═O, while the former configuration may also allow a few Fe^IV^═O formations as discussed below. In addition, the Bader charge of PMS on Fe_1_–S–Fe_1_/CN (−1.55) was larger than that of Fe_1_–Fe_1_/CN (‐1.41), confirming that Fe_1_–S–Fe_1_/CN donated more electrons to the adsorbed PMS (Figure 3f). Based on the new insights into the activation of PMS toward Fe^IV^═O production here and in previous reports on the high‐valent metal‐oxo species formation,^[^ ^8^, ^12^, ^13^, ^55^ ^]^ we focused on the free energies of each elementary step that proceeded through the evolution of two important reaction intermediates (Figure 3g). The activated *PMS was first divided into *OH and *SO_4_^−^ species via the O─O bond cleavage by the Fe_1_–S–Fe_1_/CN, followed by the transfer of the H atom to generate *Fe^IV^═O and *HSO_4_^−^ through O─H bond dissociation. Compared to Fe_1_–Fe_1_/CN, Fe_1_–S–Fe_1_/CN provided energetically favorable formation of these intermediates for Fe^IV^═O generation. The O─H bond dissociation at such an O_ter_ + O_hyd_ configuration provided a near‐zero energy barrier in the presence of bridging S, which was much lower than without S (0.74 eV), indicating that the O─H bond dissociation was spontaneous and bridging S could facilitate the selective generation of Fe^IV^═O.

### 2.4 Superior Performance of Fe^IV^═O Produced by S‐Bridged Fe Atoms

**Figure 4:**
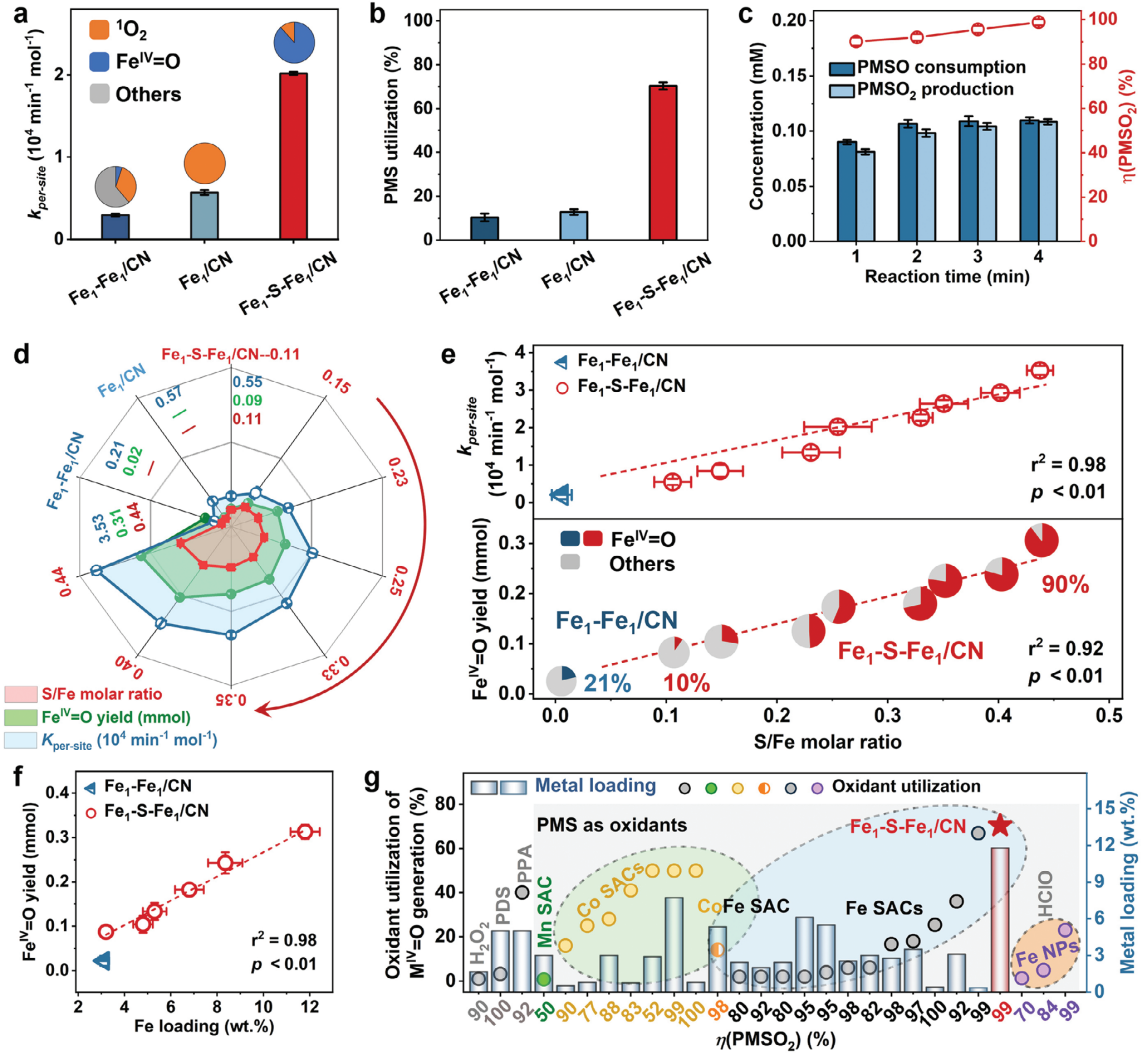
Superior performance of Fe^IV^═O produced by S‐bridged Fe atoms. a) 4‐CP degradation rates (inserted pie charts represent the contributions of reactive species) and b) PMS utilizations in different catalytic systems. c) PMSO transformation and Fe^IV^═O selectivity (indexed by the yield of PMSO_2_) in the Fe_1_–S–Fe_1_/CN/PMS system. Correlations of d) radar and e) linear graphs of *k*_per‐site_ and Fe^IV^═O yield with S content (inserted pie charts in bottom half graph represent the quantified compositions of reactive species). f) Correlation between Fe^IV^═O yield and Fe loading. g) Comparison of oxidant utilization and PMSO_2_ yield with state‐of‐the‐art catalysts.

Bridging adjacent Fe atoms with S could remarkably accelerate the degradation of *p*‐chlorophenol (4‐CP), which was used as a reactivity probe (Figures S8 and S9, Supporting Information). Considering some atoms of the high‐loading Fe atoms are inevitably located in the interior of g–C_3_N_4_ carrier. The degradation rate of 4‐CP was normalized by Fe content (*k*_per‐site_) used to rule out the impact of Fe content and to reveal the S‐induced effects via a fair comparison. The *k*_per‐site_ of Fe_1_–S–Fe_1_/CN (2.02 × 10^4^ min^−1^ mol^−1^) was 10.5 and 3.6 times higher than that of Fe_1_–Fe_1_/CN and Fe_1_/CN, respectively (**Figure** **4**a), where the catalysts possessed similar surface areas (Table S4, Supporting Information). This probably originated from the selective formation of Fe^IV^═O at a high yield. In addition, Fe_1_–S–Fe_1_/CN exhibited good pH tolerance, for example, leaching 0.49–0.68% of Fe at a pH range of 3.7–9.0 (Figure S10, Supporting Information). Although the mineralization of 4‐CP was not high (29.2%) (Figure S11, Supporting Information), Fe^IV^═O holds a promise toward higher selectivity for the targeted contaminant removal and higher utilization of PMS than radicals, forming intermediate products with relatively low toxicity via oxygen transfer reactions.^[^ ^56^ ^]^ Scavenging experiments using varied trapping agents for radicals and nonradicals were further verified, and the degradation of efficiency and the corresponding *k*_per‐site_ of Fe_1_–S–Fe_1_/CN are significantly inhibited when PMSO was added (Figure S12, Supporting Information), which suggests that the Fe_1_–S–Fe_1_/CN activates the PMS to selectively generate Fe^IV^═O, providing a 90% contribution to the 4‐CP degradation. The rest was mainly attributed to ^1^O_2_ due to the NaN_3_ and NBT also slightly inhibiting its degradation effect, and MeOH or TBA can hardly interfere with its well degradation process, suggesting the radical contributions (i.e., •OH and SO_4_^•−^) were ruled out. In contrast, the contribution of Fe^IV^═O to the relatively slow degradation of 4‐CP was only 5% in the Fe_1_–Fe_1_/CN system, in which radicals played a major role in the 4‐CP degradation due to the Fe sites in Fe_1_–Fe_1_/CN existence of the single atom, cluster, and partial nanoparticles with low loading (Figure 4a; Figure S8; Table S5, Supporting Information). These results further imply that the bridging S largely favored the selective generation of Fe^IV^═O.

Moreover, the PMS utilization was quantified as the fraction of PMS equivalents that are used to generate reactive oxygen species (Figure S13; Table S5, Supporting Information) to assess if the bridging S coordination could reduce the PMS cost.^[^ ^13^ ^]^ Notably, the PMS utilization of Fe_1_–S–Fe_1_/CN reached 70%, which was 6.7 and 5.4 times higher than that of Fe_1_–Fe_1_/CN and Fe_1_/CN, respectively (Figure 4b). This indicates that the bridging S was beneficial in enhancing the PMS utilization and accompanied by the selective generation of Fe^IV^═O at a high yield. PMSO was used to probe Fe^IV^═O because it can be selectively oxidized by Fe^IV^═O to form PMSO_2_ via an oxygen atom transfer reaction. The selectivity of Fe^IV^═O was described by the yield of PMSO_2_, i.e., *η*(PMSO_2_), which was calculated by the molar ratio of obtained PMSO_2_ toward lost PMSO.^[^ ^7^, ^14^ ^]^ The *η*(PMSO_2_) was maintained at a high level during the whole reaction and reached 99% at the end (i.e., 10 min) in the Fe_1_–S–Fe_1_/CN/PMS system (Figure 4c).

The impacts of S content and Fe loading on the yield and proportion of Fe^IV^═O by the Fe_1_–S–Fe_1_/CN were deciphered. The *k*_per‐site_ positively correlated with the S/Fe molar ratios (Figure 4d,e; Figure S14; Table S6, Supporting Information) and with the Fe^IV^═O yield (Figure 4d,e). The *k*_per‐site_ was increased from 0.55 × 10^4^ min^−1^ mol^−1^ to 3.53 × 10^4^ min^−1^ mol^−1^ and Fe^IV^═O yield was increased from 0.09 mmol to 0.31 mmol when the S/Fe molar ratio increased from 0.11 to 0.44, respectively (Figure 4d,e). The selectivity of Fe^IV^═O generation was significantly improved from 10% to 90% (the pie chart in Figure 4e), indicating the selective formation of Fe^IV^═O was controllable by simply changing the bridging S content. In addition, similar S/Fe molar ratios in Fe_1_S/CN with Fe‐S coordination and Fe_1_/SCN with C─S─C have been reported in our previous study.^[^ ^13^ ^]^ The proportion of Fe^IV^═O ranging from 27% to 65% is far below in the Fe_1_–S–Fe_1_/CN/PMS system with the same conditions. While the above characterizations and theoretical calculations of materials provided the first proof that the geometric and electronic structures of adjacent Fe atoms could be tuned by bridging S, these experimental results verified the second proof of its unique role in the selective production of Fe^IV^═O and related contaminant degradation. In addition, thanks to the bridging effect of S for preventing potential agglomeration of adjacent Fe atoms at high loadings, the yield of Fe^IV^═O was further increased to 0.31 mmol (per mol PMS) when the Fe loading increased to 11.8 wt.% (Figure 4f; Figure S15, Supporting Information). Meanwhile, the Fe_1_–S–Fe_1_/CN could give a relatively more promising PMS utilization (70%) and Fe^IV^═O selectivity (99%) than other state‐of‐the‐art catalysts for producing high‐metal valent–oxo species in advanced oxidation processes (Figure 4g; Table S7, Supporting Information).

### 2.5 Application Potential for Sustainable Water Treatments

**Figure 5:**
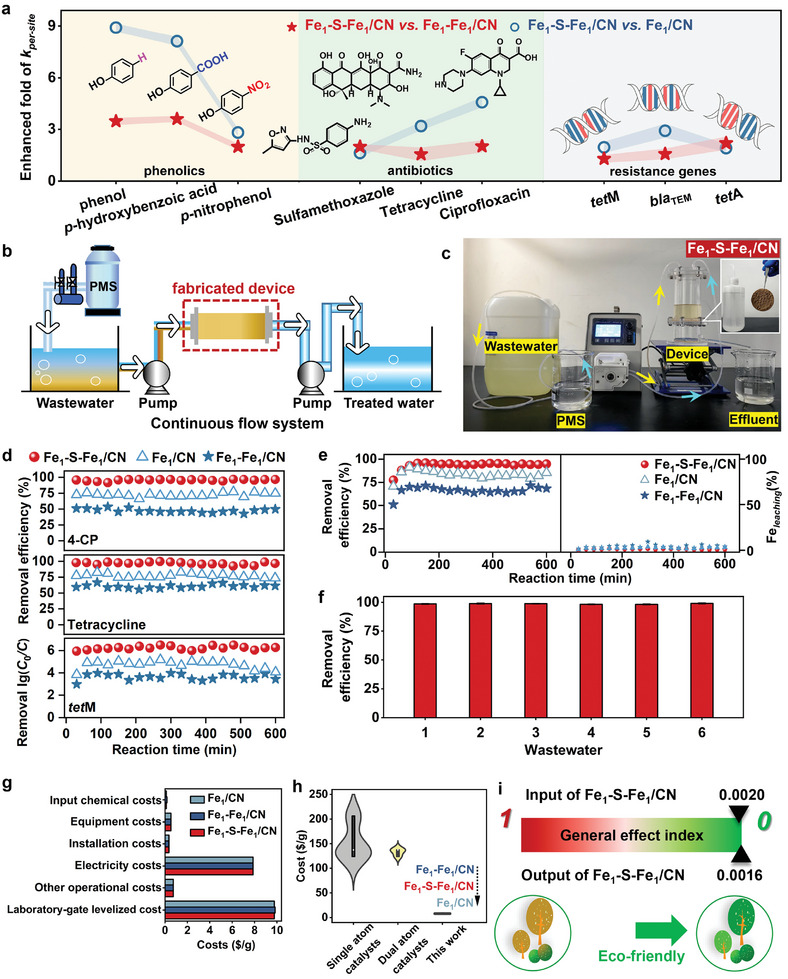
Application potential assessments of catalysts fabricated in an integrated device for sustainable water treatments. a) Enhancement of *k*_per‐site_ by Fe_1_–S–Fe_1_/CN over Fe_1_/CN and Fe_1_–Fe_1_/CN for various contaminants in batch experiments. b) Schematic of catalyst‐modified membrane fabrication and continuous flow reactor. c) Removal efficiencies of phenolics, antibiotics, and antibiotic resistance genes in continuous flow reactions. d) Removal efficiencies of typical contaminants in continuous flow reactions. e) Performance and stability of catalysts in continuous flow reactions for deep treatment of 4‐CP in wastewater. f) Removal efficiencies of 4‐CP by Fe_1_–S–Fe_1_/CN in six varied wastewaters. The technol‐economic assessments of catalysts, including g) production cost, h) cost comparison to commercial catalysts, and i) eco‐environmental feasibility of synthesis.

The application potential of these catalysts was explored for multiple contaminants, including phenolic, antibiotics, and antibiotics resistance genes (ARGs) (**Figure** **5**a; Figures S16–S18, Supporting Information). The enhancement of bridging S was universal for three different types of contaminants. The *k*_per‐site_ of multiple contaminants by Fe_1_–S–Fe_1_/CN exhibited a 2.8 – 8.9 and 1.9 – 3.4 fold enhancement over that of Fe_1_/CN and Fe_1_–Fe_1_/CN, respectively. The enhancement for different phenolics was consistent with their ionization potentials.^[^ ^57^ ^]^ The higher reactivity of Fe_1_–S–Fe_1_/CN for different antibiotics than other catalysts was likely due to the presence of functional groups (e.g., −NH_2_, −OH, and −F) in these molecules, which were readily reactive with Fe^IV^═O.^[^ ^58^, ^59^, ^60^ ^]^ Meanwhile, the antibiotics (e.g., tetracycline and ampicillin) are often accompanied by contamination of relevant ARGs (e.g., *tet*M, *tet*A, and *bla*_TEM_) with different kinds and content of bases. The Fe_1_–S–Fe_1_/CN also exhibited superior removal efficiency of ARGs, i.e., 5 logs within 8 min. These results show that our Fe_1_–S–Fe_1_/CN is highly promising for sustainable water treatment, including traditional and emerging contaminants.

The good anti‐interference ability of Fe^IV^═O toward various cations, anions, natural organic matter, and water matrices has been well‐established in previous studies.^[^ ^16^, ^61^ ^]^ Here, we present a feasible strategy for fabricating these materials as an integrated device (Figure 5b,c), assessing its application potential for practical water purifications. Considering that the dispersed catalysts in solution may cause secondary contamination and not favor their reuse, membrane technology with a bright future for supplying clean water resources was adopted.^[^ ^55^ ^]^ The long‐term stable and efficient removal of 4‐CP, tetracycline, and *tet*M by Fe_1_–S–Fe_1_/CN was good in the continuous flow systems (Figure 5d). The long‐term performance of the continuous‐flow system for degrading 4‐CP was evaluated over 600 min (20 cycles). The fabricated water‐purification filter with Fe_1_–S–Fe_1_/CN could continuously and efficiently remove the contaminants from the water (Figure 5e), without the need to clean the catalyst after a single cycle. Although the Fe_1_/CN and Fe_1_–Fe_1_/CN fabricated filters could also continuously remove 4‐CP, the removal efficiencies were much lower than that of Fe_1_–S–Fe_1_/CN. The low leaching amounts of Fe (< 4.2 wt.%) from the catalysts also suggest their good long‐term stability within 600 min, as well as constant and efficient oxidation of contaminants in aqueous solution (Figure 5e). In addition, for the purification process of the secondary effluent water, the Fe_1_–S–Fe_1_/CN reactor showed excellent treatment efficiency, indicating that the as‐prepared Fe_1_–S–Fe_1_/CN can potentially enable advanced wastewater treatment (Figure 5f; Figure S19, Supporting Information).

To evaluate the economic feasibility of this possible strategy, we conducted a preliminary technol‐economic analysis (TEA), assuming a moderate synthesis strategy the Fe SACs are thought as the final product for the TEA analysis (Text S2; Tables S8–10, Supporting Information).^[^ ^62^ ^]^ The study underscores the potential of this method to significantly reduce the production costs of high‐loading Fe SAC (Figure 5g), e.g., the laboratory‐gate levelized cost was about 9.8 $/g. This was much lower than those of the commercial single‐atom catalysts and dual‐atom catalysts (123–206 $/g), highlighting the economic viability of our synthesis strategy (Figure 5h). In addition, the possible impacts of the self‐assembly strategy on ecosystems and humans were quantitatively assessed in 14 aspects using the Biwer‐Heinzle environmental evaluation method (Tables S11–13, Supporting Information).^[^ ^57^ ^]^ The input and output general effect index (GEI) values of the self‐assembly production process were about 0.0020 and 0.0016 (Figure 5I; Figure S20, Supporting Information), indicating that the self‐assembly production process of Fe SACs is eco‐friendly to the environment.

## 3 Conclusion

In summary, the selective generation of Fe^IV^═O at a high yield was achieved by constructing adjacent Fe atoms with an S bridge. Experimental results and computational calculations together indicate that bridging S not only could increase the distance between two adjacent Fe atoms and their loading (up to 11.8 wt.%) with inhibited Fe agglomeration (being universal to other transition metals), but also could regulate the occupying electron density of *d*‐orbitals of the Fe atoms. The tunable geometric and electronic effects facilitate the proton dissociation of O_hyd_ followed by adsorption energetics on the O_bri_. Fe_1_–S–Fe_1_/CN has high PMS utilization (70%), selective generation of Fe^IV^═O (90%), Fe^IV^═O yield (63% of PMS), and Fe^IV^═O selectivity (99%). These benefits provide high reactivity, long‐term stability, and wide performance for the degradation of various traditional and emerging contaminants in both batch and continuous flow reactors. The technol‐economic assessment indicates an eco‐friendly production process with a much lower production cost of Fe SACs (9.8 $/g) than those commercial versions. This study provides a novel means to selectively produce Fe^IV^═O at a high yield via precisely constructing two adjacent Fe atoms with S bridges for water purification, and presents a great potential for catalyst‐fabricated membranes in pilot or scale‐up plants with continuous operations.

## 4 Experimental Section

### Chemicals and Reagents

All chemical reagents were of analytic grade without further purification, and the complete list with details was provided in Text S3 (Supporting Information).

### Synthesis of Fe_1_/CN, Fe_1_–Fe_1_/CN, Fe_1_–S–Fe_1_/CN, and CN Catalysts

The simple synthesis method used in this work includes two stages: namely self‐assembly and calcination (Figure 1a). In stage I, 6.4 mmol cyanuric acid (CA) and 1.6 mmol ferric citrate solution were mixed with 8 mmol melamine solution, and stirred at room temperature for 4.0 h. The solid residue was washed with deionized (DI) water and filter‐dried to obtain supermolecular powders. In stage II, the supermolecular powder (3.0 g) was heated up to 600 °C in Ar at a rate of 5 °C min^−1^ and maintained at this temperature for 4.0 h to achieve Fe_1_/CN catalyst. The Fe_1_–Fe_1_/CN and Fe_1_–S–Fe_1_/CN were synthesized in the same way by replacing ferric‐citrate solution with ferrous‐hydroxylamine hydrochloride and ferrous‐thioacetamide solution, respectively. The Fe_1_–Fe_1_/CN and Fe_1_–S–Fe_1_/CN with different Fe loadings were synthesized by changing the dosage of iron precursors while retaining the ligand dosage of 1.6 mmol (Table S1, Supporting Information), and the Fe_1_–S–Fe_1_/CN with different S/Fe molar ratios were synthesized by changing the dosage of thioacetamide while retaining the ligand dosage of 0.4 mmol (Table S6, Supporting Information). These Cu, Co, Ni, and Mn SACs were synthesized in the same way by replacing ferrous sulfate with cupric sulfate, cobalt sulfate, nickel sulfate, and manganese sulfate, respectively. In addition, CN was synthesized in the same way by changing 8 mmol CA without any Fe‐free solution from 6.4 mmol CA + 1.6 mmol ferric‐citrate solution.

### Batch Experiments

All catalytic degradation tests were carried out in a 100 mL flat‐bottom beaker at room temperature (25 ± 2 °C) with a stirring speed of 200 rpm. A specific amount of the catalysts (0.5 g L^−1^) was added to a solution containing a predetermined concentration of 4‐CP (or other contaminants), followed by ultrasonication to form a homogeneous suspension. The predetermined initial pH of the 4‐CP solution was adjusted using NaOH or H_2_SO_4_ (0.1 M). Subsequently, the suspension was stirred magnetically for 30 min to establish the adsorption‐desorption equilibrium. Quantitative peroxymonosulfate (PMS, KHSO_5_•0.5 KHSO_4_•0.5 K_2_SO_4_, 99%) was added to initiate the degradation reaction. Samples were extracted at regular intervals and immediately filtrated with a 0.22 µm polytetrafluoroethylene filter, and the concentration of 4‐CP was detected by an ultraperformance liquid chromatography (HPLC, Ultimate 3000, Thermo). ICP‐OES analyzed the leaching of Fe. All the experiments were conducted in triplicates and the average data with their standard deviations were obtained. The reaction kinetics constant rate (*k*_obs_) for the degradation of 4‐CP was analyzed using ln(*C*_t_/*C*_0_) = – *k*_obs_ × t. The per‐site *k* value (*k*_per‐site_) can be calculated using *k*_per‐site_ = *k*_obs_ *M* / (*m* × wt.%). *C*_t_ is the 4‐CP concentration at a certain reaction time (t) and *C*_0_ is the initial 4‐CP concentration (after reaching adsorption‐desorption equilibrium). *k*_obs_ is the apparent constant rate, *M* is relative atomic mass, *m* is the catalyst's mass used in the PMS‐AOPs (25 mg here), and wt.% is the metal ions content determined by ICP‐OES data.

The reaction solution (Unit: mL) was passed through a reactor at a rate of 1068 µL min^−1^ with a residence time of 30 min. The as‐prepared 0.2 g catalysts (i.e., Fe_1_/CN, Fe_1_–Fe_1_/CN, and Fe_1_–S–Fe_1_/CN) were strongly attached to the polyvinylidene difluoride membrane surface (approximately 9 cm × 9 cm) to ensure that it would not come off during the catalytic reaction. The PMS solution (0.5 mM) and simulated wastewater (e.g., 4‐CP = 0.1 mM, tetracycline = 0.1 mM, and *tet*M = 10^7^ copies/µL) were pumped separately into the continuous flow reactor (flow rate = 1068 µL min^−1^) and the leaching Fe ions content of as‐prepared catalysts (0.2 g) was determined by ICP‐MS data. Figure 5c shows the photograph of the flow reactor. The actual wastewater samples were selected from the secondary effluent of a wastewater treatment plant, and other conditions were consistent with the device experiment except that the required PMS solution was increased to 2 mM. In the universal wastewater treatment experiment, the conditions were consistent with the device experiment, except for the required solution from a wastewater treatment plant (e.g., electroplating, dyeing, domestic, and industrial).

### Characterizations

TEM and HAADF images were taken by a Tecnai F20 (working voltage: 200 kV) and a JEM‐ARM200F TEM/STEM with a spherical aberration corrector (working voltage: 300 kV), respectively. XRD analysis was done using a Bruker D8 Advance X‐ray diffractometer with Cu‐Kα radiation (λ = 0.1541 nm). FTIR was performed using a Bruker‐vector‐22 (VERTEX‐70 spectrometer). XPS analysis was captured by a Thermo Fisher Scientific (VG Escalab 250XI spectrometer with an Al anode, hν = 1486.6 eV). Raman measurements were done on a Horiba Jobin Yvon Modular Raman Spectrometer with a laser wavelength of 532 nm. The Fe and S concentration in as‐prepared catalysts measurements were conducted on the Agilent 5110 with an inductively coupled plasma optical emission spectrometer (ICP‐OES). XAS spectrum of Fe K‐edge was measured in a transmission mode at the beamline BL5S1 of Aichi Synchrotron Radiation Center Japan. Soft XAS measurement was performed at the BL08U1A beamline of the Shanghai Synchrotron Radiation Facility. The XAS analysis is represented in detail in Text S4 (Supporting Information). The relevant Cu, Co, Ni, and Mn and their normal samples were tested using RapidXAFS 2M (Anhui Absorption Spectroscopy Analysis Instrument Co., Ltd.) in transmission mode at 20 kV and 20 mA, and Si (365), Si (274), Si (238), and Si (321) spherically bent crystal analyzers with a radius of curvature of 500 mm were used. EPR spectrometer was recorded on a Bruker E500 Germany at room temperature. DMPO (50.0 mM) in MeOH and aqueous dispersion in an O_2_‐free environment was used to identify superoxide radical (O_2_^•−^), •OH, and SO_4_^•−^ radicals, respectively, while TEMP (50.0 mM) in aqueous dispersion was directly used to identify the ^1^O_2_. Detailed descriptions are shown in Texts S5 and S6 (Supporting Information).

### Analytical Methods

The concentrations of contaminants (4‐CP, ciprofloxacin, sulfamethoxazole, tetracycline, p‐nitrophenol, phenol, hydroxybenzoic acid, BQ, PMSO, and PMSO_2_) were monitored by the HPLC with a TC‐C18 reverse‐phase column (150 mm × 4.6 mm, 5 µm, Agilent). The detailed analysis methods are described in Table S14 (Supporting Information). The measurement of PMS concentration, iron species, and contaminants and the qualitative analysis of reactive oxygen species (i.e., •OH, O_2_^•−^, SO_4_^•−^, Fe^IV^═O, and ^1^O_2_) are described in Text S5 (Supporting Information). The scavenging experiments were performed by adding to MeOH, TBA, NBT, PMSO, and NaN_3_, which were calibrated by the molar ratio with PMS to distinguish the participation of •OH, O_2_^•−^, SO_4_^•−^, Fe^IV^═O, and ^1^O_2_, respectively. The reaction rate constants after adding tert‐butanol (TBA), MeOH, nitroblue tetrazolium (NBT), and PMSO were denoted as *k*_1_, *k*_2_, *k*_3_, and *k*_4_, respectively, and the initial rate constant without a quenching agent was *k*_0_. The contributions of •OH, O_2_^•−^, SO_4_^•−^, Fe^IV^═O, and ^1^O_2_ were calculated with the methods detailed in Text S6 (Supporting Information).

### Computational Details

All the spin‐polarized Born‐Oppenheimer DFT calculations were conducted using the Vienna Ab initio Simulation Package (VASP) with the projector augmented wave (PAW) method to describe the inner orbital electrons.^[^ ^63^ ^]^ The Kohn‐Sham (K‐S) wave functions were expanded using a plane wave basis set with a 500 eV cutoff energy to treat the valence electrons. The revised Perdew‐Burke‐Ernzerhof (RPBE) functional of the generalized gradient approximation (GGA) was utilized to assess the exchange‐correlation component of the K‐S equation throughout.^[^ ^64^ ^]^ The Grimme's D3 (DFT‐D3) method with the zero‐damping function was applied for the dispersion correction.^[^ ^65^ ^]^ The self‐consistent electronic loop was converged to 10^−5^ eV and the structural models were considered to be at equilibrium when the system force was less than 0.03 eV Å^−1^. A 3 × 3 × 1 *k*‐point mesh based on the Monkhorst‐Pack method was used to sample the Brillouin zone for the geometric calculations.^[^ ^66^ ^]^ We used the IBRION = 5 option in VASP with a 0.02 Å atomic displacement to calculate the vibrational modes of reaction intermediates. The Bader charge analysis was performed for the electron transfer. Further, a denser 5 × 5 × 1 Monkhorst‐Pack *k*‐point mesh was used to obtain an accurate density of states (DOS). The details of adsorption energy and charge density difference are described in Text S7 (Supporting Information). To investigate the mechanism of Fe^IV^═O generation, the details of catalyst models and standard Gibbs free energy of PMS activation reaction are described in Text S8 (Supporting Information).

## Conflict of Interest

The authors declare no conflict of interest.

## Author Contributions

X.J. and Z.G. contributed equally to this work. X.J. and J.X. conceived and designed the project. X.J., Z.G., Z.P., and Y.C. performed the experiments. Z.G., C.M., H.L., H.O., Y.C., and G.H. conducted the theoretical calculations and analysis tools. X.J., J.X., X.X., L.Z., and D.L. analyzed the data. X.J., J.X., and D.L. co‐wrote and revised the manuscript. All authors provided critical feedback and assisted during manuscript preparation.

## Supporting information



Supporting Information

## Data Availability

The data that support the findings of this study are available from the corresponding author upon reasonable request.
