# Supplementary material for: Sulfur Bridge Geometry Boosts Selective FeIV═O Generation for Efficient Fenton‐Like Reactions
Source: Adv Sci (Weinh). 2025 Mar 5;12(16):2500313. doi: 10.1002/advs.202500313 (PMC12021096; doi:10.1002/advs.202500313)
Supplement: Supplementary file 1 — Supporting Information [file ADVS-12-2500313-s001.pdf]

ADVANCED SCIENCE
Open Access

Supporting Information

for *Adv. Sci.*, DOI 10.1002/advs.202500313

Sulfur Bridge Geometry Boosts Selective $Fe^{IV}=O$ Generation for Efficient Fenton-Like Reactions

Xunheng Jiang, Zhongyuan Guo, Jiang Xu, Zhiyu Pan, Chen Miao, Yue Chen, Hao Li, Hiroshi Oji, Yitao Cui, Graeme Henkelman, Xinhua Xu, Lizhong Zhu and Daohui Lin**

Supporting Information

Sulfur Bridge Geometry Boosts Selective $Fe^{IV}=O$ Generation for Efficient Fenton-like Reactions

Xunheng Jiang,† Zhongyuan Guo,† Jiang Xu, Zhiyu Pan, Chen Miao, Yue Chen, Hao Li, Hiroshi Oji, Yitao Cui, Graeme Henkelman, Xinhua Xu, Lizhong Zhu, Daohui Lin**

X. H. Jiang, Z. Y. Guo, J. Xu, Z. Y. Pan, C. Miao, Y. Chen, X. H. Xu, L. Z. Zhu, D. H. Lin
College of Environmental and Resource Sciences, Zhejiang University, Hangzhou 310058, China
E-mail: xujiang6@zju.edu.cn. lindaohui@zju.edu.cn

X. H. Jiang, J. Xu, L. Z. Zhu, D. H. Lin

Zhejiang Provincial Key Laboratory of Organic Pollution Process and Control, Zhejiang University, Hangzhou 310058, China

H. Li
Advanced Institute for Materials Research (WPI-AIMR), Tohoku University, Sendai, Japan

H. Oji
Synchrotron Radiation Research Center, Nagoya University, Nagoya, Japan

Y. T. Cui
Institute of Advanced Science Facilities, Shenzhen, China

G. Henkelman
Department of Chemistry and the Oden Institute for Computational Engineering and Sciences, University of Texas at Austin, Austin, USA

† These authors contributed equally to this work.

3 Sections, 14 Tables, 20 Figures

Table of Contents

Experimental Procedures 3
1. Measurement and Analysis of NN Distance Distribution between Fe Atoms 3
2. Techno-Economic Assessment of the Ligand-Assisted Supermolecular Self-Assembly Strategy to Produce Fe_1/CN, Fe_1–Fe_1/CN, and Fe_1–S–Fe_1/CN 3
3. Complete List of Chemicals 4
4. XAS Analysis Methods 5
5. Measurements of the Active Species and PMS 5
6. Calculating the Contribution of Active Species by Quenching Tests 6
7. Adsorption energy and charge density difference 6
8. Catalyst models and standard Gibbs free energy of reaction 7
Figures and Tables 8
References 44

Experimental Procedures.

Text S1. Measurement and analysis of NN distance distribution between Fe toms

To assess the dispersion of Fe atoms on the as-prepared catalysts, the nearest neighbor (NN) distance distributions between Fe atoms were compared to the measured and theoretical (random dispersion).[1,2] Specifically, the enhancement of the atom contrast in AC–HAADF–STEM images of each sample via a Laplacian–of–Gaussian filter. Then further analyses in AC–HAADF–STEM images that the Gaussians and the center of Gaussians were fitted as the single Fe atom and set as the Fe atom locations, respectively. The areal density (marked as λ) was eventually calculated by counting the number of Fe atoms in the selected images and dividing the corresponding area. If all Fe atoms were randomly deposited on the surface of the CN carrier, the locations of Fe atoms would be analyzed by a Poisson random field, and the probability density functions (PDF) for the distribution of NN distances distribution between Fe atoms (marked as r) should follow a Rayleigh distribution as Eq. S1.[3,4]

$$\mathrm{PDF}(r) = 2\pi\lambda r \times \exp(\pi\lambda r^2) \tag{S1}$$

where the mean value of the distribution was given by $(\lambda^{-1/2})/2$. We can also evaluate the degree of clustering of Fe atoms in each catalyst by comparing the experimental NN distances to Rayleigh distribution.

Text S2. Techno-economic assessment of the ligand-assisted supermolecular self-assembly strategy to produce Fe_1/CN, Fe_1–Fe_1/CN, and Fe_1–S–Fe_1/CN.

To evaluate the economic potential of scalable synthesis of Fe SACs (i.e., Fe_1/CN, Fe_1–Fe_1/CN, and Fe_1–S–Fe_1/CN), the technol-economic assessment of the ligand-assisted supermolecular self-assembly strategy was analyzed via the calculated model of the laboratory-gate levelized cost.[5,6] The laboratory-gate levelized cost can be divided into input chemicals, equipment, installation, electricity, and other operations. The detailed calculation is as follows:

Input chemical cost. The chemical prices were adjusted according to experimental production or literature reported (Table S8). The chemical proportion was referred to as gram-level synthesis. The Ar flow and flow time were set to 0.1 mL/min and 10 h. The input chemical cost can be calculated as Eq. S2 below.

$$\text{Input chemical cost [\$ per tonne of material]} = \sum \text{all chemicals price} \tag{S2}$$

Equipment cost. According to the production process of Fe SAC, the costs of purchasing equipment were calculated. The detailed parameters and the price of equipment as shown in

Table S9. Purchasing equipment can be converted to the cost of the product one gram of Fe SAC. We assume the lifetime (~20 years), a laboratory capacity factor (~0.9), and a discount rate (i) (~7%) of that equipment and no salvage value at the end of the laboratory's time. The capital recovery factor (CRF) can be calculated as Eq. S3 below.

$$CRF_{equipment}=\frac{i(1+i)^{lifetime}}{(1+i)^{lifetime}-1}=\frac{0.07(1+0.07)^{20}}{(1+0.07)^{20}-1}=0.094 \quad (S3)$$

The equipment cost per gram of Fe SAC can be calculated as Eq. S4 below.

$$\text{Equipment cost [\$ per tonne of material]}=\frac{CRF_{equipment}\times \text{total equipment price [\$]}}{\text{capacity factor}\times 365\ \text{day}\times \text{production capacity}\left[\frac{t}{day}\right]} \quad (S4)$$

Installation cost. We assume a Lang factor of 1 for the equipment installation cost and the total capital cost is equal to the equipment cost as Eq. S5 below.

$$\text{Electricity cost [\$ per tonne of material]}=\sum \text{all equipment power consumed [kW]}\times 24\ \text{[h]}\times \text{electricity price}\left[\frac{\$}{kWh}\right] \quad (S5)$$

Other operational cost. Other operational costs (i.e., labor, maintenance, and waste treatment) are assumed to be 10% of the total capital costs calculated above. The other operational cost can be calculated as Eq. S6 below.

Other operational cost =10%*(Input chemical cost + Equipment cost + Installation cost) (S6)

Laboratory-gate levelized cost. The laboratory-gate levelized cost of producing one gram of Fe SAC can be calculated by summing up all the costs mentioned above as Eq. S7 below.

Laboratory-gate levelized cost = Input chemical cost + Equipment cost + Installation cost + Other operational cost (S7)

Text S3. Complete list of chemicals.

Unless otherwise specified, all chemicals were analytically pure. Iron(III) nitrate nonahydrate ($Fe(NO_3)_3$•H_2O, 98.5%, AR) was purchased from Macklin Co., Ltd. Melamine (MA, $C_3H_6N_6$, 99%, AR), cyanuric acid (CA, $C_3H_3N_3O_3$, 98%, AR), ferrous sulfate heptahydrate ($FeSO_4$•$7H_2O$, 99%, AR), citric acid (CTA, $C_6H_8O_7$, 99.5%, AR), hydroxylamine hydrochloride (HH, NH_3OHCl, 99%, AR), Thioacetamide (TCA, C_2H_5NS, 99%, ACS), *p*-chlorophenol (4-CP, C_6H_5OCl, 99%, AR), sulfamethoxazole ($C_{10}H_{11}N_3O_3S$, 98%, AR), tetracycline ($C_{22}H_{24}N_2O_8$, AR, 98%), ciprofloxacin ($C_{17}H_{18}FN_3O_3$, 99%, AR), hydroxybenzoic

acid ($C_7H_6O_3$, 98%, AR), terephthalic acid (TA, $C_8H_6O_4$, 99%, AR), cobalt chloride hexyahydrate ($CoCl_2{\bullet}6H_2O$, AR), potassium peroxymonosulfate (PMS, $KHSO_5{\bullet}0.5 KHSO_4{\bullet}0.5 K_2SO_4$, 99%), 5,5-dimethyl-1-pyrroline-N-oxide (DMPO), 2,2,6,6-tetramethyl piperidinyloxyl (TEMP), methyl phenyl sulfoxide (PMSO, 99%, AR), methyl phenyl sulfone ($PMSO_2$, 99%, AR), methyl alcohol (MeOH, CH_3OH, AR), ethanol (C_2H_5OH, AR), and tert-butanol (TBA, $C_4H_{10}O$, AR) were purchased from Aladdin Co., Ltd. Sodium azide (NaN_3, AR) and 2,2-azino-bis(3-ethylbenzonthiazoline)-6-sulfonic acid diammonium (ABTS, 99%) were purchased from Sigma-Aldrich. Nitroblue tetrazolium (NBT, AR) and *p*-benzoquinone (BQ, AR) were purchased from J&K Scientific. All aqueous solutions were prepared using deionized (DI) water with a resistivity of 18.2 $M\Omega{\bullet}cm^{-1}$.

Text S4. XAS analysis methods

The X-ray beam was carried out in ionization chamber transmission mode using water-cooled Si (111) double-crystal monochromators, and was focused with two Rh-coated focusing mirrors with a beam size of 2.0 mm in the horizontal direction and 0.5 mm in the vertical direction around sample position to obtain extended X-ray adsorption fine structure (XAFS) spectra both in the near and extended edge. The samples were made in a pellet of 10 mm diameter. The Fe foil, FeO, Fe_2O_3, Fe_3O_4, and FeS samples were used as references, and all samples were measured by transmission mode. The spectra were analyzed and fitted using an analysis program Demeter and in the R space with a k-weight of 3. Wavelet simulation was performed with using the continuous Cauchy wavelet transform method. For EXAFS fittings, the crystal structure of metal foil was from the Materials Project.[7]

Text S5. Measurements of the active species and PMS

The residual concentration of PMS was determined by an ABTS method.[8] The qualitative analysis of •OH was identified by the terephthalic acid photoluminescence (TA-PL) technique.[9] The production of $O_2^{\bullet-}$ and $^1O_2$ were examined using the NBT method and the 1,3-diphenylisobenzofuran (DPBF) method.[10,11] The quantitation of $SO_4^{\bullet-}$ and $Fe^{IV}{=}O$ were determined by BQ originating from $SO_4^{\bullet-}$-derived HBA and $PMSO_2$ originating from $Fe^{IV}{=}O$-derived PMSO via HPLC analysis.[12,13] We further quantified the levels of •OH, $SO_4^{\bullet-}$, $O_2^{\bullet-}$, $Fe^{IV}{=}O$, and $^1O_2$, and calculated the PMS utilization using $PMS_{utilization}(\%) = \sum(ROS) / C(PMS_{comsuption})$ during the reaction processes.

Text S6. Calculating the contribution of active species by quenching tests

We quantitively determined the contributions from •OH, $SO_4^{\bullet -}$, $O_2^{\bullet -}$, Fe^{IV}=O, and $^1O_2$. After adding TBA, MeOH, NBT, PMSO, and NaN_3, the reaction rate constants (k_{obs}) or per-site k value ($k_{per\text{-}site}$) were denoted as k_1, k_2, k_3, k_4, and k_5, respectively, and the initial rate constant without a quenching agent was k_0. The contributions (λ) of •OH, $SO_4^{\bullet -}$, $O_2^{\bullet -}$, Fe^{IV}=O, and $^1O_2$ were calculated according to Eqs. S8−13, respectively.

$$\lambda(\bullet OH) = [(k_0 - k_1) / k_0] \times 100\% \tag{S8}$$

$$\lambda(SO_4^{\bullet -}) = [(k_1 - k_2) / k_0] \times 100\% \tag{S9}$$

$$\lambda(O_2^{\bullet -}) = [(k_0 - k_3) / k_0] \times 100\% \tag{S10}$$

$$\lambda(Fe^{IV}{=}O) = [(k_0 - k_4) / k_0] \times 100\% \tag{S11}$$

$$\lambda(^1O_2) = [(k_0 - k_5) / k_0] \times 100\% \tag{S12}$$

$$\lambda(^1O_2 \text{ or } Fe^{IV}{=}O) = 1 - \lambda(\bullet OH) - \lambda(SO_4^{\bullet -}) - \lambda(O_2^{\bullet -}) - \lambda(Fe^{IV}{=}O \text{ or } ^1O_2) \tag{S13}$$

where $\lambda(\bullet OH)$, $\lambda(SO_4^{\bullet -})$, $\lambda(O_2^{\bullet -})$, $\lambda(Fe^{IV}{=}O)$, and $\lambda(^1O_2)$ were the contribution of •OH, $SO_4^{\bullet -}$, $O_2^{\bullet -}$, Fe^{IV}=O, and $^1O_2$ to degradation of 4-CP, respectively. For Fe_1–S–Fe_1/CN reaction system, $\lambda(Fe^{IV}{=}O) = [(k_0 - k_4) / k_0] \times 100\%$ and $\lambda(^1O_2) = 1 - \lambda(\bullet OH) - \lambda(SO_4^{\bullet -}) - \lambda(O_2^{\bullet -}) - \lambda(Fe^{IV}{=}O)$; For Fe_1/CN and Fe_1–Fe_1/CN reaction systems, $\lambda(^1O_2) = [(k_0 - k_5) / k_0] \times 100\%$ and $\lambda(Fe^{IV}{=}O) = 1 - \lambda(\bullet OH) - \lambda(SO_4^{\bullet -}) - \lambda(O_2^{\bullet -}) - \lambda(^1O_2)$.

Text S7. Adsorption energy and charge density difference

The PMS group has three different oxygen (O) atoms (O_{ter}, O_{bri}, and O_{hyd}), and different adsorption configurations on Fe sites lead to different oxidative mechanisms of contaminants. The adsorption energy (E_{ads}) of PMS on catalysts was defined in Eq. S14 below:

$$E_{ads} = E_{total} - E_{cat.} - E_{PMS} \tag{S14}$$

where E_{total}, $E_{cat.}$ and E_{PMS} are the DFT-electronic energies of the catalyst–PMS composite, the catalyst (cat.), and the PMS group, respectively.

The charge density difference induced by PMS adsorption was calculated using Eq. S15 below:

$$\Delta\rho = \rho(*PMS) - \rho(*) - \rho(PMS) \tag{S15}$$

where ρ(*PMS), ρ(*), and ρ(PMS) are three different charge density quantities. When calculating the latter two quantities, the atomic positions are fixed as those in the *PMS composite.

Text S8. Catalyst models and standard Gibbs free energy of reaction

To reveal the performance origin induced by bridge-S dopants on iron (Fe)-doped g-C_3N_4, three tri-s-triazine-based g-C_3N_4 (t-C_3N_4)-based catalyst models were built according to our experiment characterizations: two Fe atoms with/without a bridge-S atom were doped into a (2 × 2) supercell of t-C_3N_4 based on the synchrotron characterization analysis results; as a comparison, the single-atom Fe catalyst, doped into a (2 × 2) supercell of t-C_3N_4, was also built. To reduce the impact of periodic images on the model slab in the *z*-direction, a 20 Å vacuum thickness was added perpendicular to the slab in that direction.

To uncover the production mechanism of Fe^{IV}=O species, the reaction-free evolution for the PMS decomposition on catalysts was determined using the computational hydrogen electrode (CHE) model.[14] The Gibbs free energy of the proton-electron couple (H^+/e^-) can be written as in Eq. S16 below:

$$G^0(H^+ + e^-) = 1/2 \cdot G^0H_2 - e \cdot U \quad \text{(S16)}$$

where G^0H_2 is the standard Gibbs free energy of H_2, and *U* is the external applied bias.

For an elementary reaction, * + ($H^+ + e^-$) + A ↔ *HA, where * denotes the catalyst site or catalyst, A denotes the reactant, and HA denotes the reaction intermediate. The standard Gibbs free energy change can be calculated as in Eq. S17 below:

$$\Delta G^0 = G^0{*HA} - G^0A - G^0{*} - G^0(H^+ + e^-) \quad \text{(S17)}$$

Furthermore, the standard Gibbs free energy of a reaction intermediate R (*R) can be calculated as in Eq. S18 below:

$$G^0_{*R} = E^{DFT}{}_R + E_{ZPE,\,R} - T \cdot S_{vib,\,R} \quad \text{(S18)}$$

Where $E^{DFT}{}_R$ is the DFT energy of the optimized intermediate at 0 K, $E_{ZPE,\,S}$ is the vibrational zero-point energy (ZPE), T is the reaction temperature (set to 298.15 K), and $S_{vib,\,R}$ is the vibrational entropy (*S*) of the adsorbed species on the slab surface, R (*R).

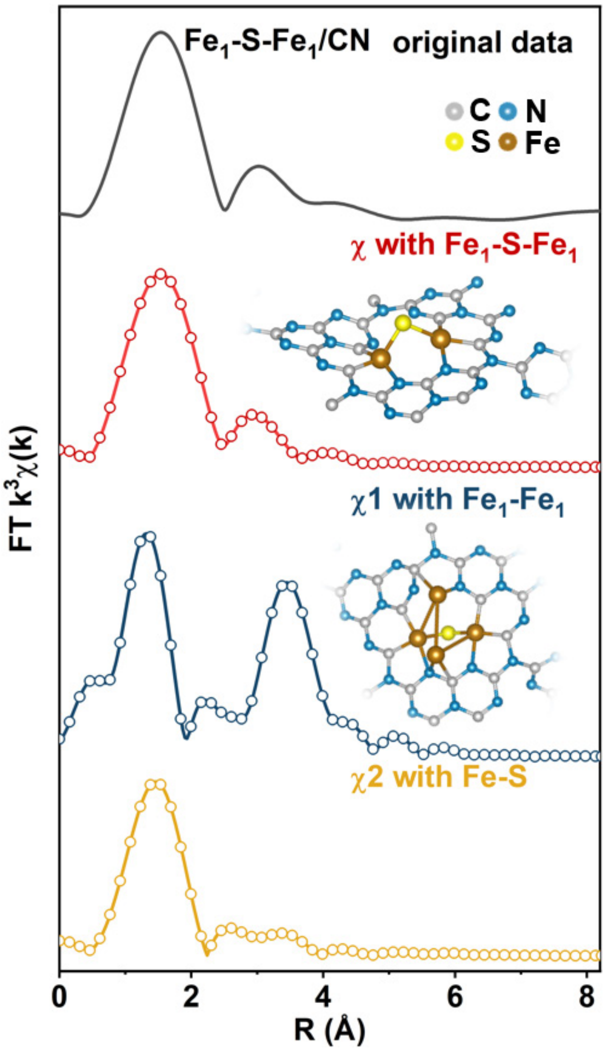

Figure S1. Possible EXAFS fitting paths in *R* space of Fe_1–S–Fe_1/CN.

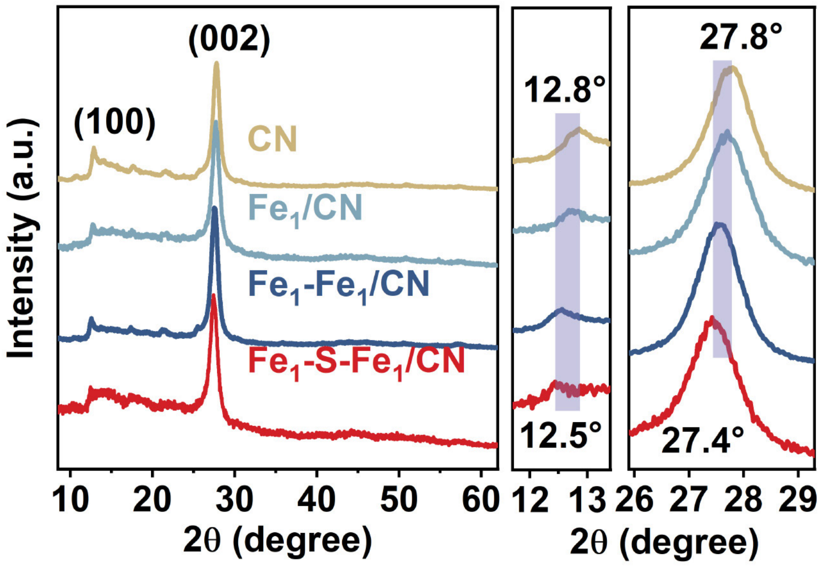

Figure S2. X-ray diffraction patterns of CN, Fe_1/CN, Fe_1–Fe_1/CN, and Fe_1–S–Fe_1/CN.

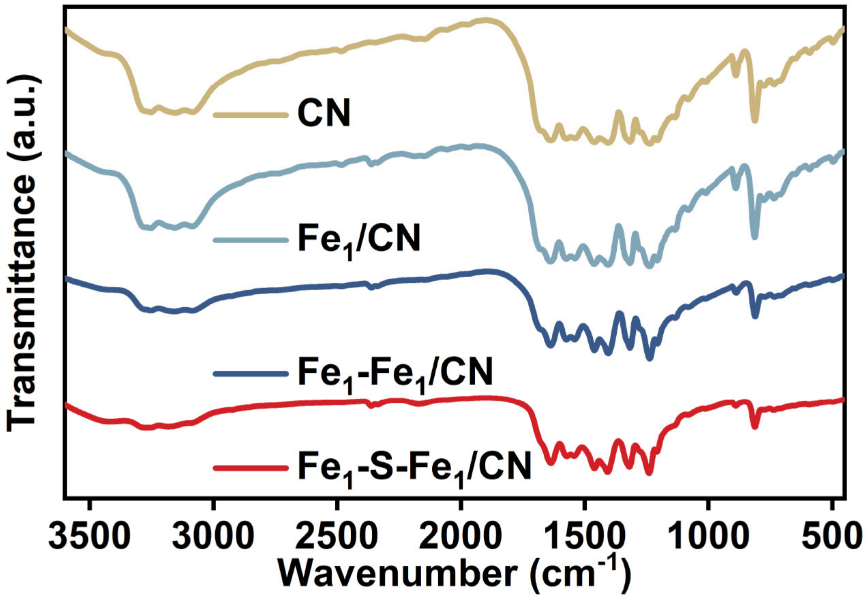

Figure S3. Fourier-transformed infrared spectra of CN, Fe_1/CN, Fe_1–Fe_1/CN, and Fe_1–S–Fe_1/CN.

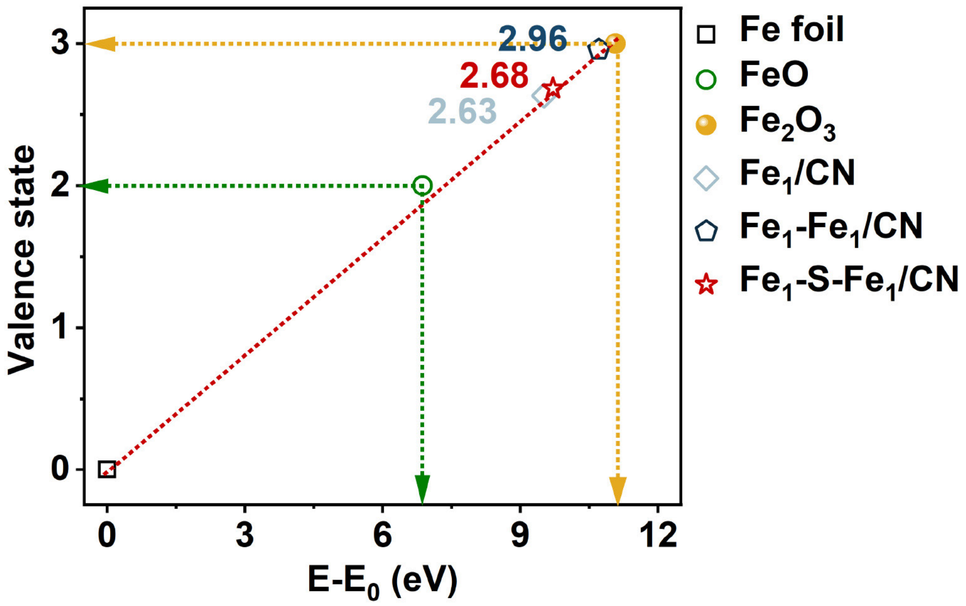

Figure S4. Fe valence state was calculated as the first derivation of as-prepared catalysts and corresponding references on XANES.

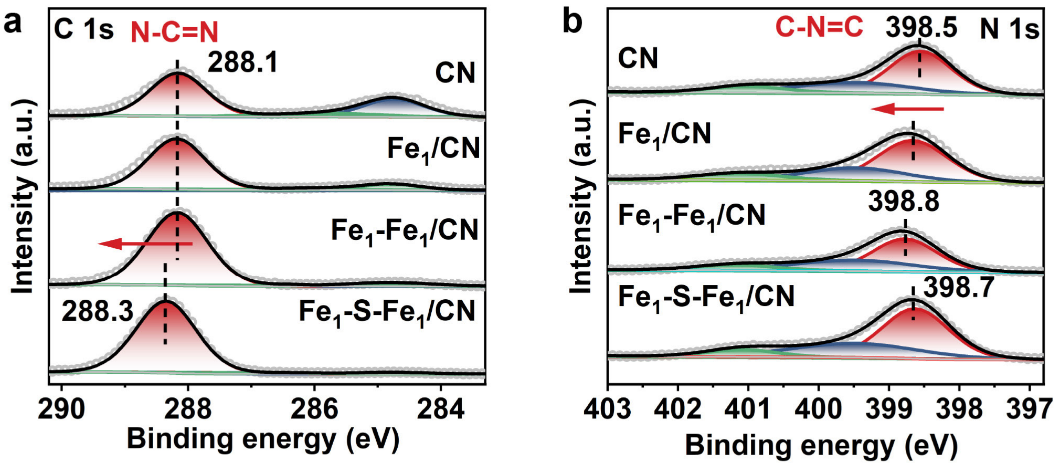

Figure S5. High-resolution of (a) C 1s and (b) N 1s XPS spectra of CN, Fe_1/CN, Fe_1–Fe_1/CN, and Fe_1–S–Fe_1/CN.

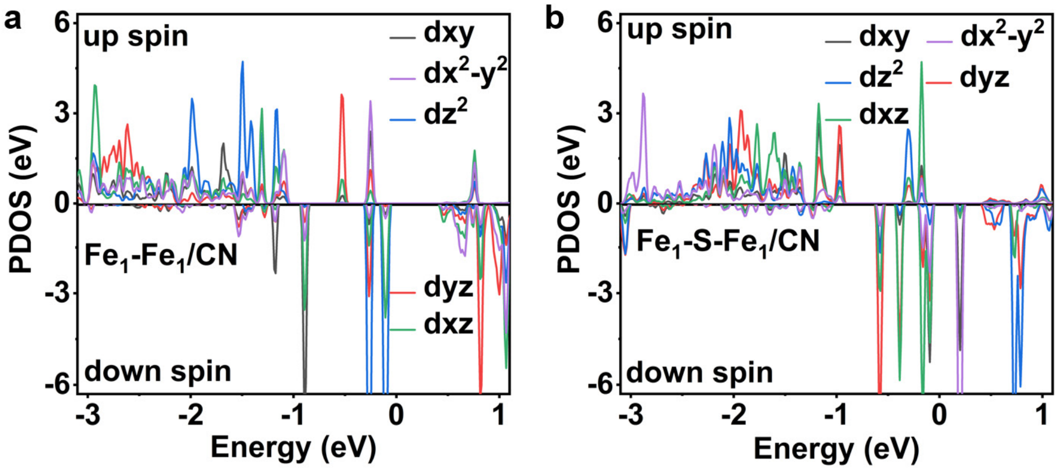

Figure S6. PDOS of Fe 3*d* orbitals in (a) Fe_1–Fe_1/CN and (b) Fe_1–S–Fe_1/CN.

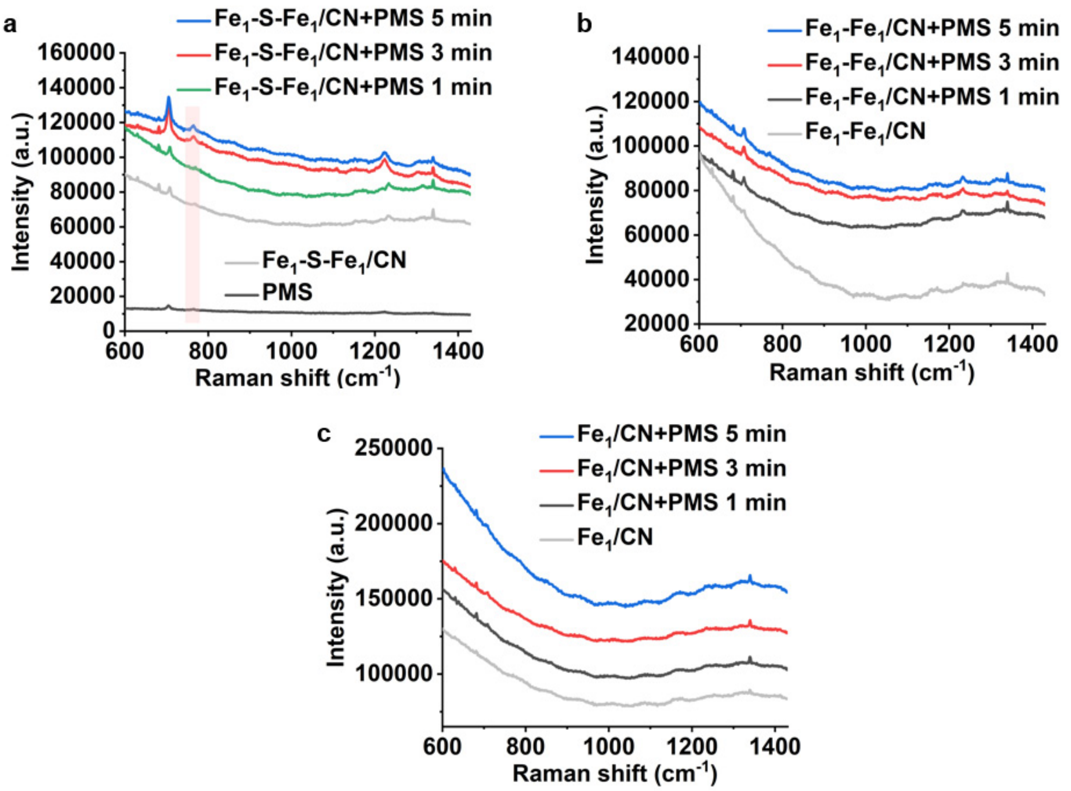

Figure S7. Raman spectra in the (a) Fe_1–S–Fe_1/CN/PMS, (b) Fe_1/CN/PMS, and (c) Fe_1–Fe_1/CN/PMS systems. (Conditions: initial pH = 5.5, T = 25 ± 2°C, 4-CP = 0.1 mM, PMS = 0.5 mM, catalysts = 0.5 g L^{-1})

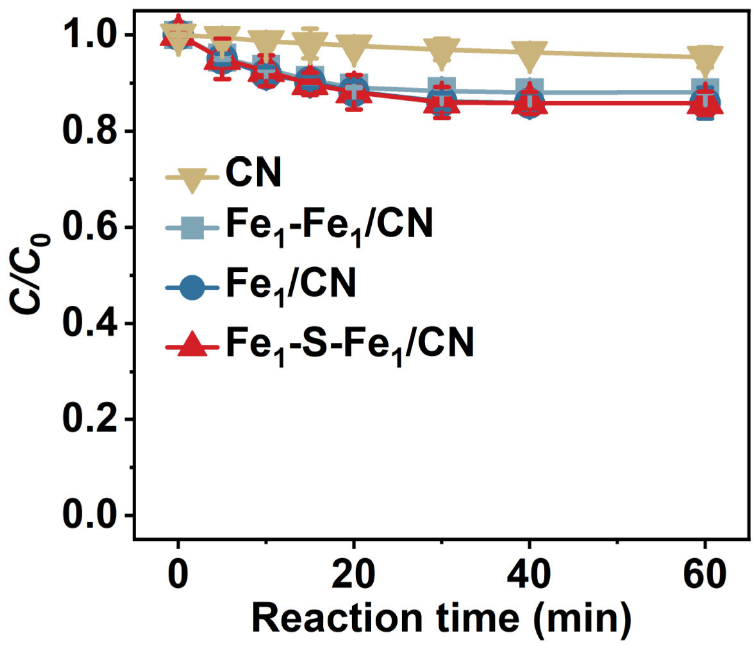

Figure S8. The adsorption efficiency of 4-CP by different catalysts in the absence of PMS. (Conditions: T = 25 ± 2°C, 4-CP = 0.1 mM, catalysts = 0.5 g L^{-1}).

Note: The adsorption of 4-CP by CN and these catalysts was relatively limited compared with their degradation in the presence of PMS.

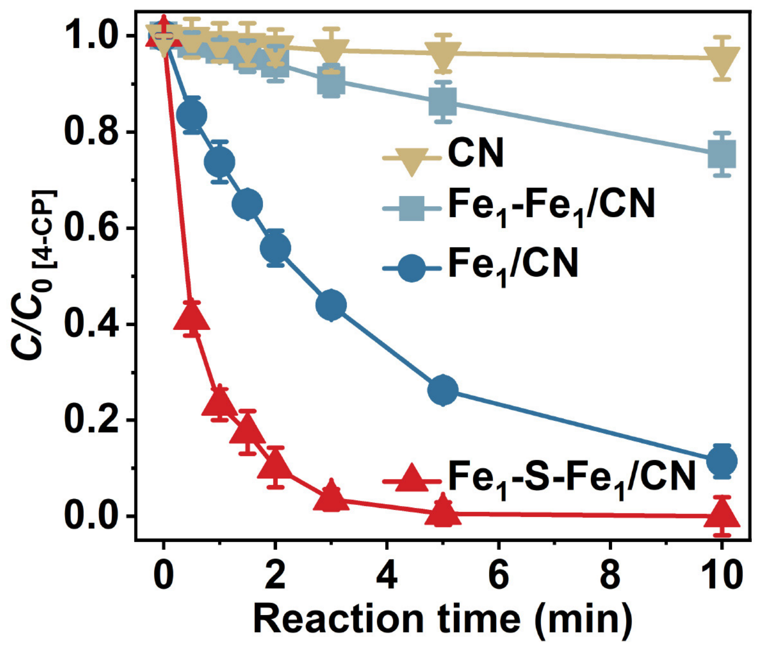

Figure S9. The 4-CP degradation efficiencies of different catalysts in the presence of PMS. (Conditions: initial pH = 5.5, T = 25 ± 2°C, 4-CP = 0.1 mM, PMS = 0.5 mM, catalysts = 0.5 g L^{-1}).

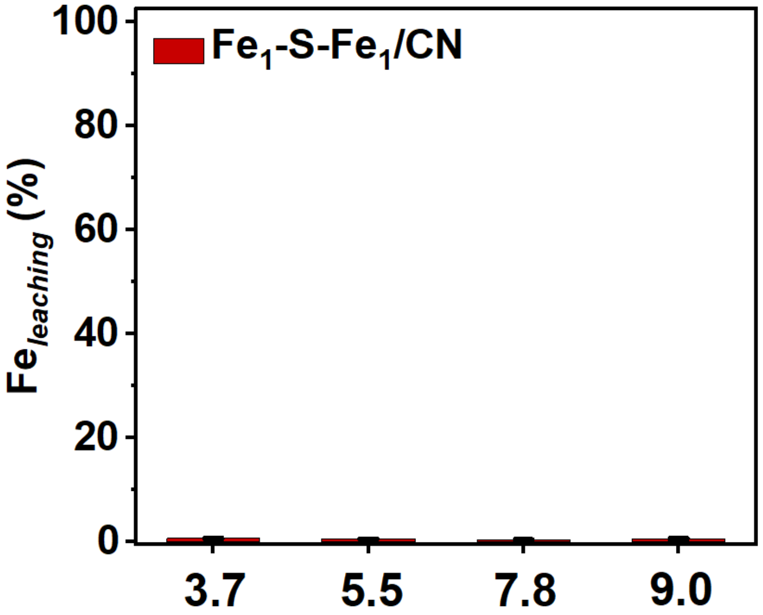

Figure S10. Fe leaching of Fe_1–S–Fe_1/CN activated PMS for 4-CP within 30 min. (Control conditions: initial pH = 3.7, 5.5, 7.8, and 9.0, T = 25 ± 2°C, 4-CP = 0.1 mM, PMS = 0.5 mM, catalysts = 0.5 g L^{-1}).

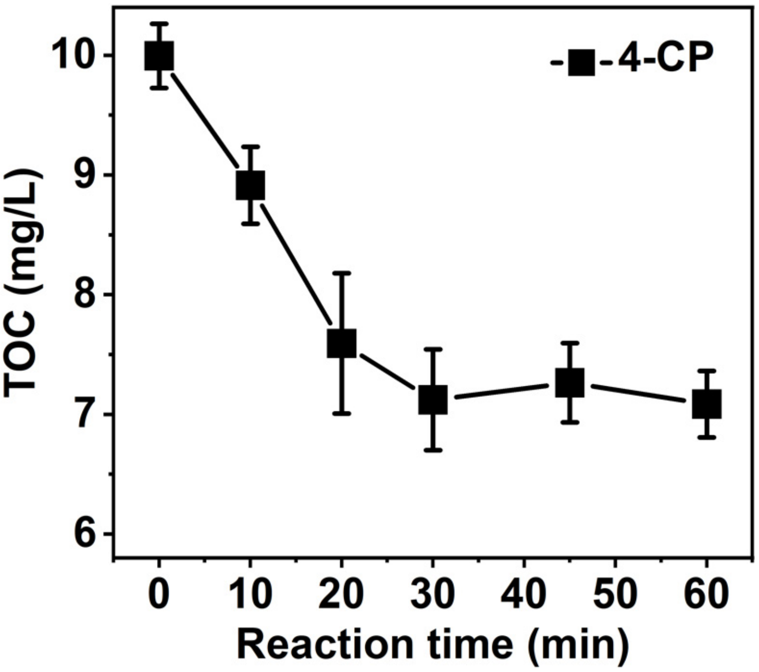

Figure S11. Changes of total organic carbon (TOC) during the 4-CP degradation in Fe_1–S–Fe_1/CN/PMS system. (Conditions: initial pH = 5.5, T = 25 ± 2°C, concentration = 0.1 mM, 0.5 mM PMS, catalysts = 0.5 g L^{-1}).

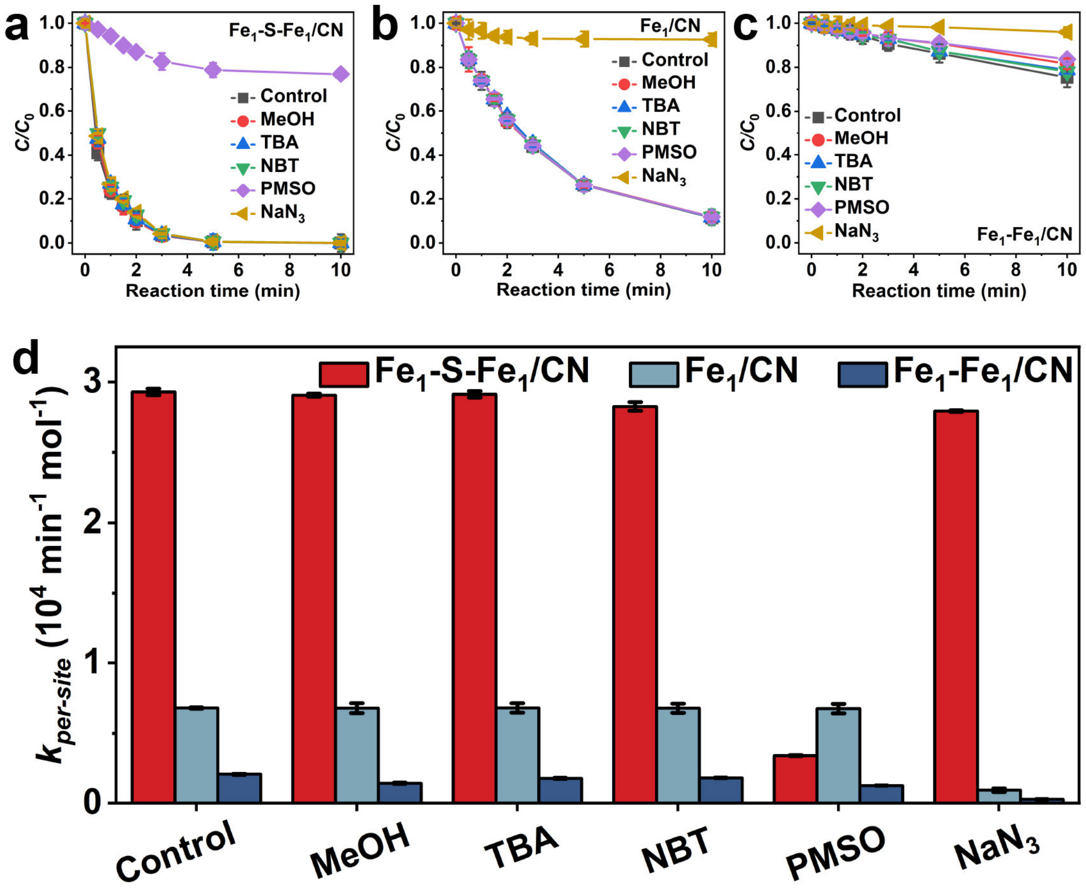

Figure S12. (a-c) 4-CP degradation efficiency and (d) $k_{per\text{-}site}$ of scavenging experiments. •OH, $SO_4^{\bullet-}$, $O_2^{\bullet-}$, Fe^{IV}═O, and $^1O_2$ were quenched by adding tert-butanol (TBA), methanol (MeOH), nitroblue tetrazolium (NBT), methyl phenyl sulfoxide (PMSO), and sodium azide (NaN_3), respectively. (Conditions: initial pH = 5.6, T = 25 ± 2°C, 4-CP = 0.1 mM, PMS = 0.5 mM, catalysts = 0.5 g L^{-1}, MeOH = TBA = 500 mM, NBT = PMSO = 2.5 mM, NaN_3 = 5 mM).

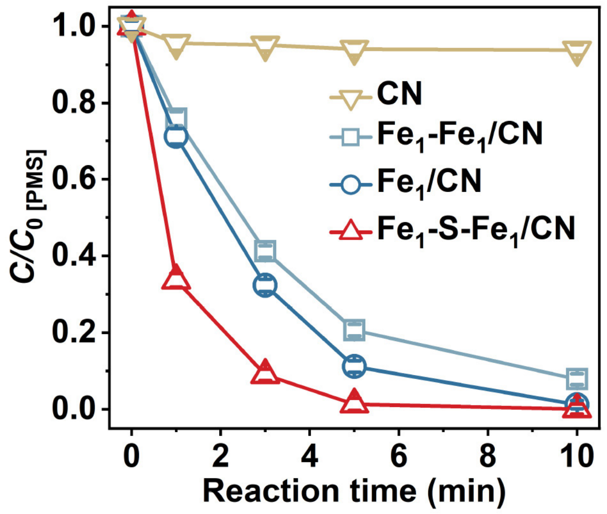

Figure S13. PMS consumption of 4-CP removal by different catalysts in the presence of PMS. (Conditions: initial pH = 5.5, T = 25 ± 2°C, 4-CP = 0.1 mM, catalysts = 0.5 g L^{-1}).

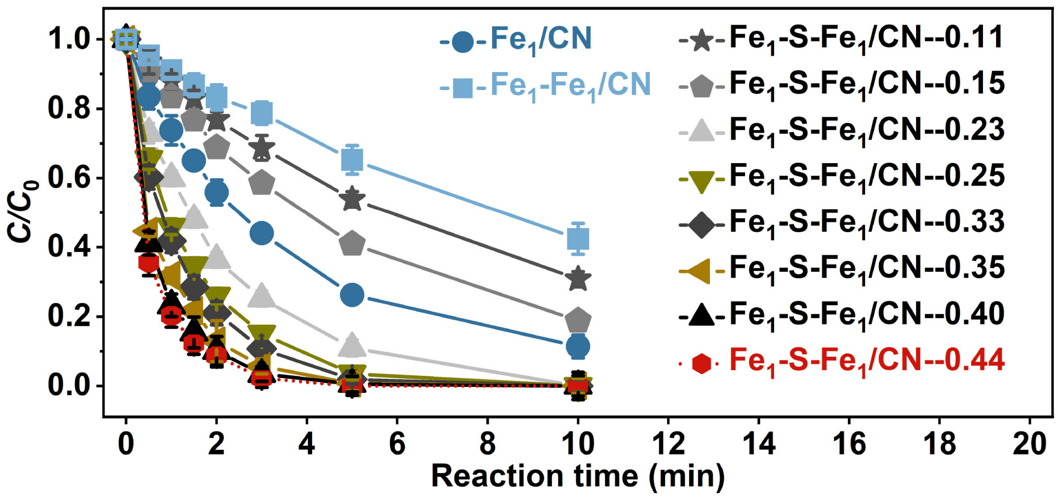

Figure S14. The 4-CP degradation efficiencies of Fe_1–Fe_1/CN, Fe_1/CN, and Fe_1–S–Fe_1/CNs with different S/Fe molar ratios in the activated PMS system. (Conditions: initial pH = 5.5, T = 25 ± 2°C, 4-CP = 0.1 mM, PMS = 0.5 mM, catalysts = 0.5 g L^{-1}).

Note: Fe_1–S–Fe_1/CN--Y with different S/Fe molar ratios were synthesized and Y is the S/Fe molar ratio of Fe_1–S–Fe_1/CNs as shown in Table S6.

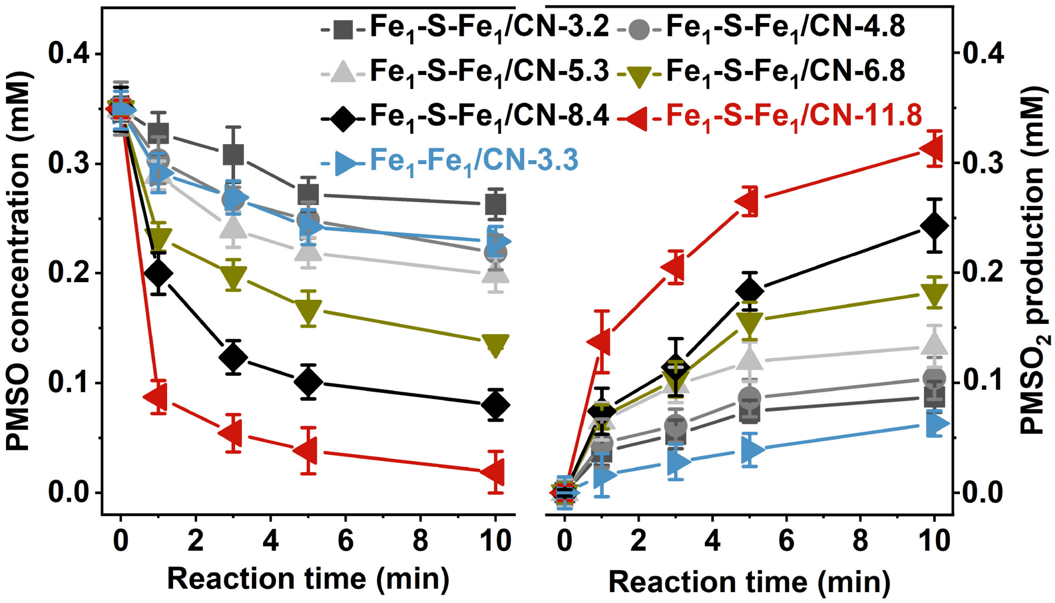

Figure S15. PMSO and $PMSO_2$ concentration in Fe_1–Fe_1/CN and Fe_1–S–Fe_1/CNs with different Fe loadings in activated PMS systems as a function of reaction time. (Conditions: initial pH = 5.5, T = 25 ± 2°C, 4-CP = 0.1 mM, PMS = 0.5 mM, catalysts = 0.5 g L^{-1}).

Note: Fe_1–Fe_1/CN-X and Fe_1–S–Fe_1/CN-X with different Fe loading were synthesized and X is the Fe content of as-prepared catalysts as shown in Table S1.

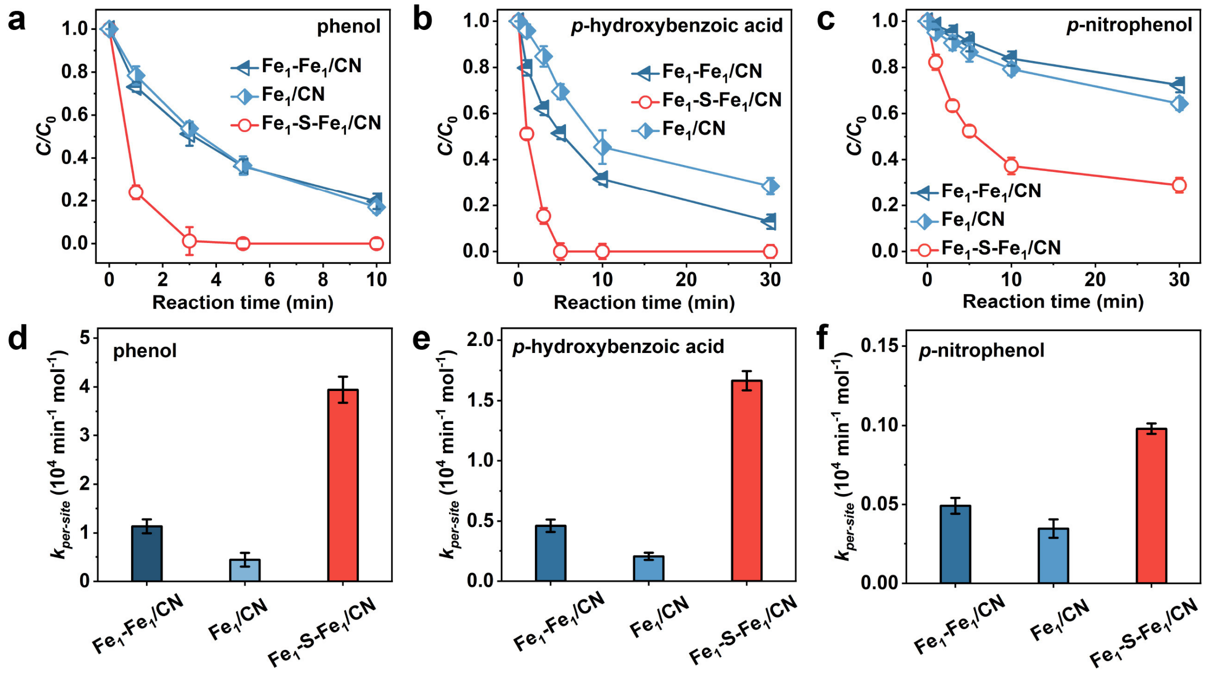

Figure S16. (a) Phenol degradation efficiencies and (d) corresponding $k_{\text{per-site}}$ of the as-prepared catalysts activated PMS; (b) *p*-hydroxybenzoic acid degradation efficiencies and (e) corresponding $k_{\text{per-site}}$ of the as-prepared catalysts activated PMS; (c) *p*-nitrophenol degradation efficiencies and (f) corresponding $k_{\text{per-site}}$ of the as-prepared catalysts activated PMS. (Conditions: initial pH = 5.6, T = 25 ± 2°C, concentration = 0.1 mM, PMS = 0.5 mM, catalysts = 0.5 g L^{-1}).

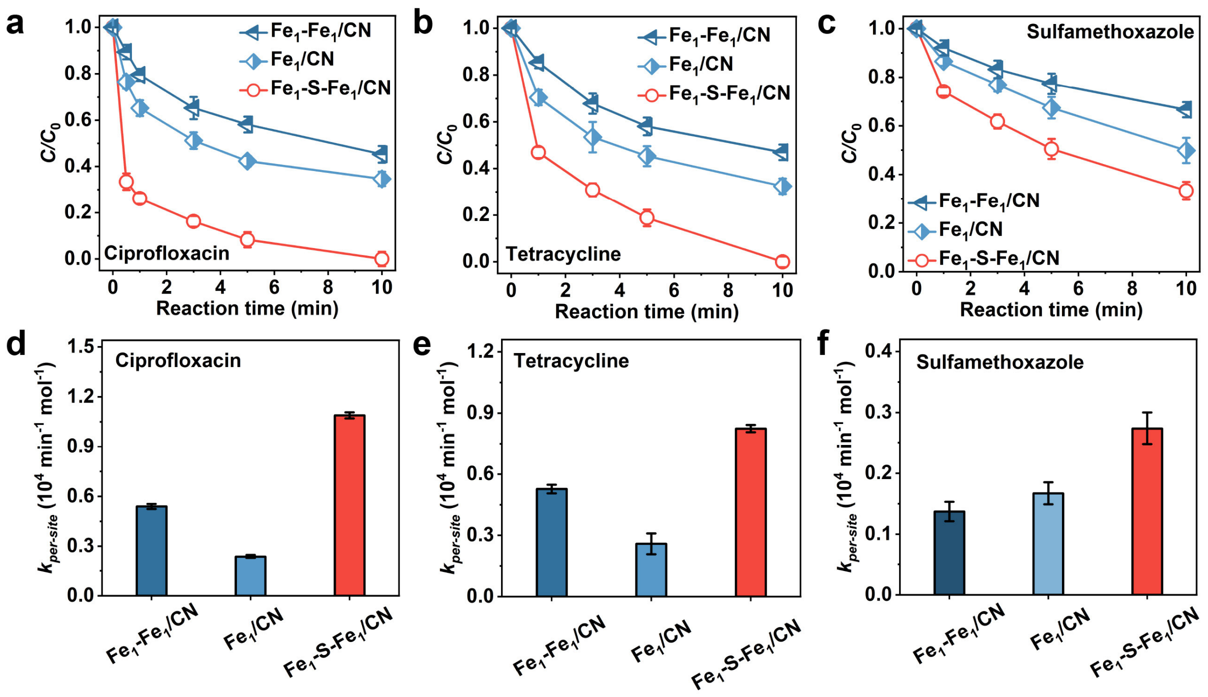

Figure S17. (a) Ciprofloxacin degradation efficiency and (d) corresponding $k_{\text{per-site}}$ by as-prepared catalysts activated PMS; (b) tetracycline degradation efficiency and (e) corresponding $k_{\text{per-site}}$ by as-prepared catalysts activated PMS; (c) sulfamethoxazole degradation efficiency and (f) corresponding $k_{\text{per-site}}$ by as-prepared catalysts activated PMS. (Conditions: initial pH = 5.6, T = 25 ± 2°C, concentration = 0.1 mM, PMS = 0.5 mM, catalysts = 0.5 g L^{-1}).

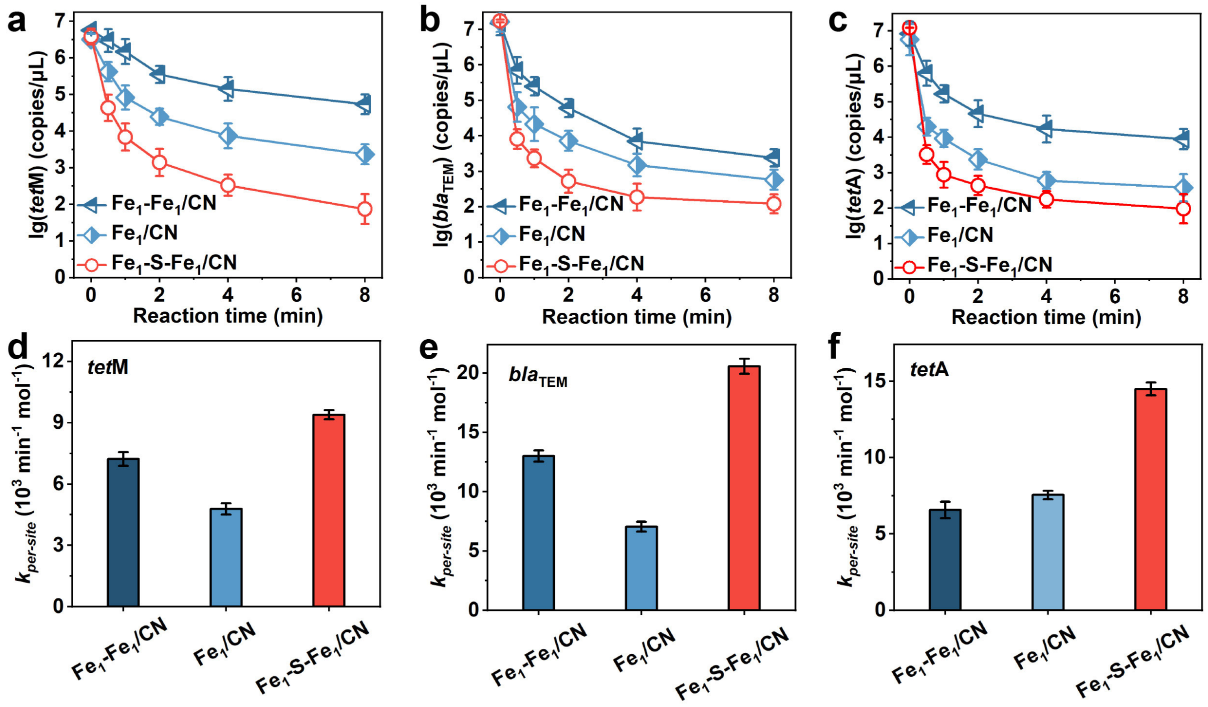

Figure S18. (a) *tet*M degradation efficiency and (d) corresponding $k_{\text{per-site}}$ by as-prepared catalysts activated PMS; (b) *bla*$_{\text{TEM}}$ degradation efficiency and (e) corresponding $k_{\text{per-site}}$ by as-prepared catalysts activated PMS; (c) *tet*A degradation efficiency and (f) corresponding $k_{\text{per-site}}$ by as-prepared catalysts activated PMS. (Conditions: initial pH = 5.6, T = 25 ± 2°C, eARGs = 10^7 copies/μL, PMS = 0.5 mM, catalysts = 0.5 g L^{-1}).

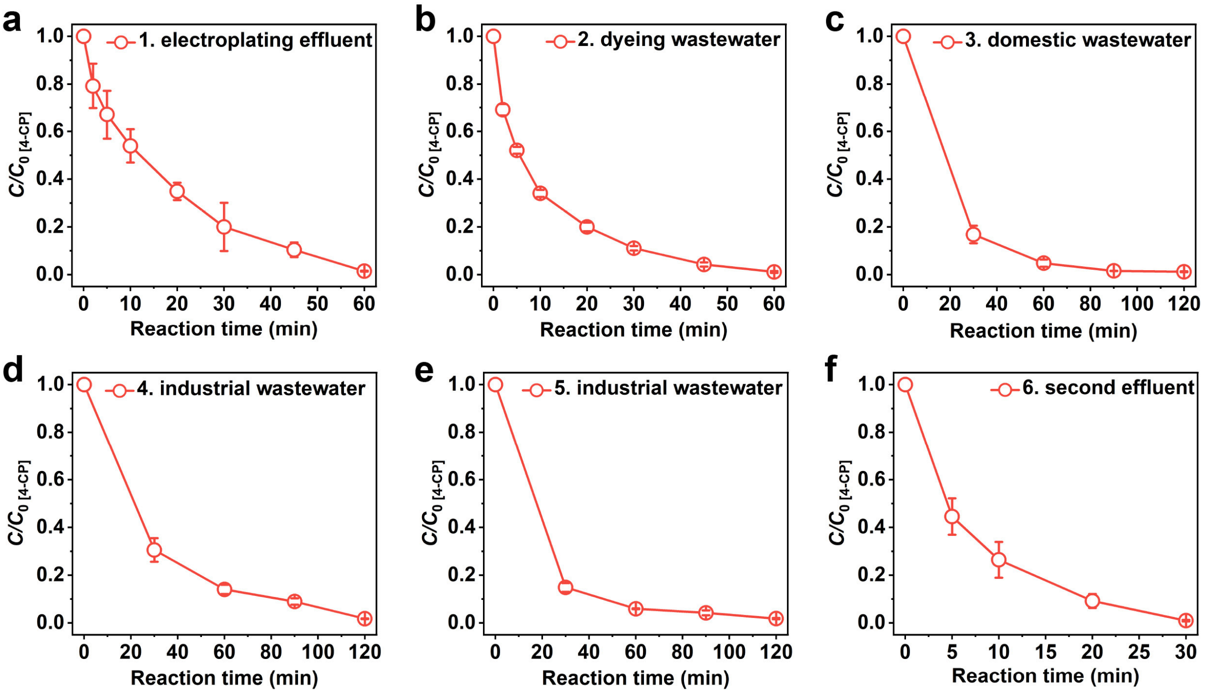

Figure S19. 4-CP degradation efficiency in actual wastewater (a)1. electroplating effluent, (b)2. dyeing wastewater, (c)3. domestic wastewater, (d)4. industrial wastewater, (e)5. industrial wastewater, (f)6. second effluent water by the Fe_1–S–Fe_1/CN reactor (Conditions: 4-CP = 0.05 mM, PMS = 0.5 mM, catalyst = 0.5 g L^{-1}).

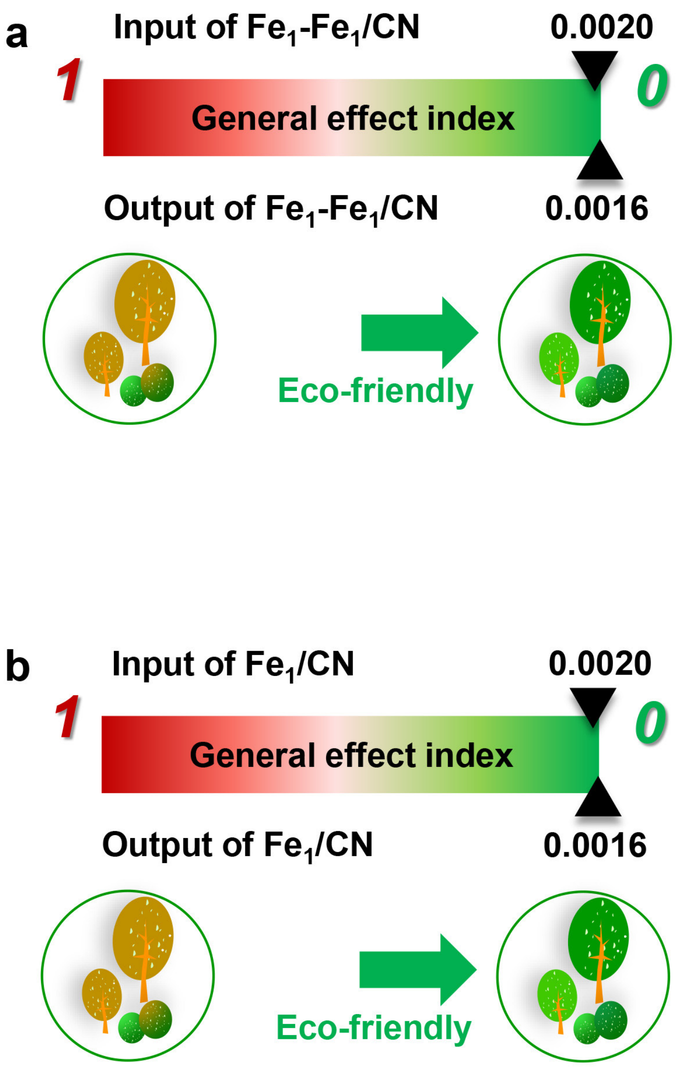

Figure S20. The general effect index of input and output of (a) Fe_1–Fe_1/CN and (b) Fe_1/CN production process.

Table S1. Fe and S contents of the as-prepared catalysts are based on different dosages of Fe source.

Samples	Fe content [wt.%][a]	S content [wt.%][b]	Fe/S molar ratio	Fe content in raw materials [wt.%][a]
Fe$_1$–S–Fe$_1$/CN-3.2	3.15±0.28	2.84±0.35	0.58±0.08	0.28±0.06
Fe$_1$–S–Fe$_1$/CN-4.8	4.76±0.42	2.86±0.43	0.97±0.06	0.55±0.11
Fe$_1$–S–Fe$_1$/CN-5.3	5.27±0.51	2.81±0.46	1.08±0.06	0.82±0.15
Fe$_1$–S–Fe$_1$/CN-6.8	6.79±0.62	2.88±0.48	1.36±0.11	1.10±0.23
Fe$_1$–S–Fe$_1$/CN-8.4	8.38±0.74	2.93±0.45	1.64±0.11	1.36±0.30
Fe$_1$–S–Fe$_1$/CN-11.8	11.79±0.63	2.98±0.48	2.27±0.25	2.02±0.48
Fe$_1$–S–Fe$_1$/CN-11.9	11.88±0.23	2.95±0.35	2.31±0.32	2.18±0.36
Fe$_1$–S–Fe$_1$/CN-12.0	11.96±0.33	2.97±0.45	2.31±0.35	2.81±0.40
Fe$_1$–S–Fe$_1$/CN-11.9	11.93±0.27	2.96±0.39	2.31±0.38	3.52±0.30
Fe$_1$–S–Fe$_1$/CN-11.9	11.89±0.35	2.93±0.43	2.33±0.42	4.38±0.34
Fe$_1$/CN-8.9	8.85±0.35	—	—	1.36±0.35
Fe$_1$–Fe$_1$/CN	2.85±0.27	—	—	0.25±0.10
	2.93±0.30	—	—	0.56±0.20
	3.05±0.46	—	—	0.76±0.43
	3.10±0.42	—	—	1.07±0.44
	3.25±0.36	—	—	1.36±0.34
	3.30±0.31	—	—	2.02±0.35

a-b: The Fe and S content of as-prepared samples confirmed by ICP-OES.

Note: Fe$_1$–Fe$_1$/CNs and Fe$_1$–S–Fe$_1$/CN-X with different Fe loadings were synthesized by changing the dosage of the iron precursors while retaining the ligand dosage of 1.6 mmol. And X (wt.%) is the Fe content in as-prepared catalysts. The maximum Fe loading was about 11.96 wt.% via continuously increasing the dosage of $FeSO_4$•$7H_2O$ in the raw material while retaining the thioacetamide dosage of 1.6 mmol.

Table S2. EXAFS fitting parameters at the Fe *K*-edge for Fe_1–S–Fe_1/CN, Fe_1–Fe_1/CN, and Fe_1/CN.

Sample	Shell	N^a	$R(Å)^b$	$\sigma^2(Å^2)^c$	*R* factor
Fe_1–S–Fe_1/CN-χ	Fe–N	1.4	2.07	0.010	0.003
	Fe–S	0.5	2.35	0.010	
	Fe–C	2.2	2.58	0.010	
Fe_1–S–Fe_1/CN-χ_1	Fe–N	1.4	1.58	0.010	0.202
	Fe–S	0.5	1.64	0.010	
	Fe–C	2.2	2.17	0.010	
	Fe–Fe	3.8	3.68	0.010	
Fe_1–S–Fe_1/CN-χ_2	Fe–N	5.0	1.59	0.010	0.001
	Fe–S	1.0	2.24	0.010	
	Fe–C	11.0	1.76	0.010	
	Fe–Fe	1.0	3.56	0.010	
Fe_1-Fe_1/CN	Fe–N	4.00	2.10	0.006	0.006
	Fe-Fe	0.98	2.75	0.006	
Fe_1/CN	Fe–N	4.00	2.10	0.009	0.006

[a]*N*: coordination numbers; [b]*R*: bond distance; [c]σ^2: Debye-Waller factors; *R* factor: goodness of fit.

Table S3. Adsorption energies (E_{ads}) of PMS on Fe_1–S–Fe_1/CN, Fe_1–Fe_1/CN, and Fe_1/CN.

Samples	Absorbed O species of PMS	Absorption sites of catalysts	Base (eV)	*PMS (eV)	E_{ads} (eV)
Fe_1–S–Fe_1/CN	O_{ter} + O_{ter}	Fe site	-456.028	-494.075	-2.69
	O_{bri} + O_{ter}		-456.028	-494.284	-2.90
	O_{hyd} + O_{ter}		-456.028	-473.801	-4.39
Fe_1–Fe_1/CN	O_{ter} + O_{ter}	Fe site	-450.550	-489.137	-3.23
	O_{bri} + O_{ter}		-450.550	-489.284	-3.36
	O_{hyd} + O_{bri} + O_{ter}		-450.550	-495.774	-6.22
Fe_1/CN	O_{ter}	Fe site	-460.386	-499.116	-3.37
	O_{bri}		-460.386	-499.289	-3.55
	O_{hyd}		-460.386	-500.144	-4.40

Table S4. The surface areas pore volumes, and pore sizes of the as-prepared samples.

Samples	Surface area ($m^2 \cdot g^{-1}$)	Pore Volume ($cm^3 \cdot g^{-1}$)	Pore Size (nm)
CN	69.3	0.34	23.1
Fe_1/CN	93.1	0.36	18.3
Fe_1–S–Fe_1/CN	102.8	0.42	19.2
Fe_1–Fe_1/CN	77.1	0.32	19.5

Table S5. Produtcion of various active species in as-prepared various systems.

Substrates	•OH (mM)	$^{\bullet}SO_4^-$ (mM)	$^{\bullet}O_2^-$ (mM)	$^1O_2$ (mM)	Fe^{VI}=O (mM)
Fe_1/CN	–	–	–	0.121	–
Fe_1–S–Fe_1/CN--0.11	0.011	–	0.017	0.068	0.025
Fe_1–S–Fe_1/CN--0.15	0.008	0.003	0.008	0.053	0.066
Fe_1–S–Fe_1/CN--0.23	0.006	0.006	0.008	0.044	0.141
Fe_1–S–Fe_1/CN--0.25	0.008	0.006	0.006	0.038	0.172
Fe_1–S–Fe_1/CN--0.33	0.003	0.003	0.003	0.069	0.202
Fe_1–S–Fe_1/CN--0.35	0.006	0.003	0.006	0.058	0.266
Fe_1–S–Fe_1/CN--0.40	0.006	0.003	0.006	0.055	0.309
Fe_1–S–Fe_1/CN--0.44	0.003	–	0.003	0.028	0.313
Fe_1–Fe_1/CN	0.014	0.012	0.009	0.032	0.022

Note: Fe_1–S–Fe_1/CN--Y with different S/Fe molar ratios were synthesized and Y is the S/Fe molar ratio of Fe_1–S–Fe_1/CNs as shown Table S6

Table S6. Fe and S contents of the as-prepared catalysts are based on different S/Fe molar ratios.

Samples	Fe content [wt.%][a]	S content [wt.%][b]	S/Fe molar ratio
Fe_1–S–Fe_1/CN--0.11	10.80±0.35	0.66±0.25	0.11±0.04
Fe_1–S–Fe_1/CN--0.15	10.94±0.12	0.94±0.33	0.15±0.05
Fe_1–S–Fe_1/CN--0.23	11.03±0.26	1.47±0.35	0.23±0.05
Fe_1–S–Fe_1/CN--0.25	11.20±0.33	1.65±0.44	0.25±0.06
Fe_1–S–Fe_1/CN--0.33	11.23±0.24	2.14±0.31	0.33±0.04
Fe_1–S–Fe_1/CN--0.35	11.35±0.15	2.30±0.37	0.35±0.05
Fe_1–S–Fe_1/CN--0.40	11.59±0.53	2.69±0.44	0.40±0.05
Fe_1–S–Fe_1/CN--0.44	11.78±0.37	2.98±0.31	0.44±0.03
Fe_1–Fe_1/CN	10.80±0.35	0.66±0.25	0.11±0.04

a-b: The Fe and S content of as-prepared samples confirmed by ICP-OES.

Note: Fe_1–S–Fe_1/CN--Y with different S/Fe molar ratios were synthesized by changing the dosage of thioacetamide while retaining the ferrous sulfate dosage of 0.4 mmol. And Y is the S/Fe molar ratio in Fe_1–S–Fe_1/CNs

Table S7. The comparison of recently representative-reported catalysts with our work.

Samples	PMS Utilization (%)	Fe^{IV}=O (mmol)	$\eta(PMSO_2)$ (%)	Refs
**Fe_1–S–Fe_1/CN**	**70.3**	**0.31**	**98.8**	**This work**
Fe-SA/PHCNS	18.0	0.0027	97.2	Ref. S15
Fe_1-CN-BDA0.75	2.0	0.0005	95.0	Ref. S16
Fe1.0-CN-TA2.0	2.0	0.001	80.0	Ref. S17
FeSANs-800	2.0	0.002	91.6	Ref. S18
ZnFe-LDH	1.3	0.0017	84.0	Ref. S19
Fe–N–C	36.0	0.00062	92.0	Ref. S20
FeSA-NPS	6.2	0.01	82.4	Ref. S21
Fe–N–C	3.3	0.00033	100	Ref. S22
FeSA–N–CNT	12.5	0.005	71.4	Ref. S23
FeSA–MCN	25.4	0.033	100	Ref. S24
FeCo–N/C	14.2	0.046	98	Ref. S25
NZVI	5.0	0.0025	99	Ref. S26
FeNx	23.0	0.023	70	Ref. S27
Fe–N_2 SACs	66.9	0.669	99.4	Ref. S28

Table S8. Input chemical amount and costs of one gram Fe SAC production.

Chemicals	Fe_1/CN			Fe_1–Fe_1/CN			Fe_1–S–Fe_1/CN		
	Price ($/g)	Amount (g)	Total cost ($)	Price ($/g)	Amount (g)	Total cost ($)	Price ($/g)	Amount (g)	Total cost ($)
$C_3H_6N_6$	0.02	3.0	0.06	0.02	3.0	0.06	0.02	3.0	0.06
$C_3H_3N_3O_3$	0.01	2.4	0.024	0.01	2.4	0.024	0.01	2.4	0.024
$C_2H_2O_4$	0.03	0.45	0.015						
$Fe(NO_3)_3$•$9H_2O$	0.013	0.48	0.006						
NH_3OHCl	–	–	–	0.254	0.33	0.084			
$FeSO_4$•$7H_2O$	–	–	–	0.009	0.33	0.003	0.009	0.33	0.003
C_2H_5NS	–	–	–				0.058	0.36	0.021
Ar	0.757 ($/L)	0.06 L	0.045	0.757 ($/L)	0.06 L	0.045	0.757 ($/L)	0.06 L	0.045
Water	0.0004 ($/L)	0.75 L	0.0003	0.0004 ($/L)	0.75 L	0.0003	0.0004 ($/L)	0.75 L	0.0003

Table S9. Capacity, electricity power, and price of equipment used in the TEA analysis.

Equipment	Capacity	Electricity power (kW)	Price ($)
Water bath	4.5 L/h	1.2	52.27
Magnetic stirrer	1.125 L/h	0.18	77.03
Tube furnace	2.0 L/h	12.0	1237.96
Drying oven	20 L/h	1.75	343.88
Extraction filtration	10 L/h	0.18	89.41

Table S10. Parameters and class limits of the impact categories. In each category, literature cited indicates possible sources for relevant data. I = Category used to evaluate input components, O = Category used to evaluate output components.

Impact category	I/O	Class A	Class B	Class C
1. Raw Materials Availability	I	only fossil predicted exhaustion within 30 years	only fossil predicted exhaustion within 30-100 years	exclusively renewable, or guaranteed long-term supply (> 100 years)
2. Complexity of the Synthesis	I	> 10 stages	3-10 stages	< 3 stages
3. Critical Material Used	I	critical materials or produced in stoichiometric amounts	critical materials involved in sub-stoichiometric amounts	no critical compounds involved
4. Thermal Risk	I/O	R 1-4, 9, 12, 15-17, 44	R 5-8, 10, 11,14, 18, 19, 30	–
5. Acute Toxicity	I/O	R 26-28, 32	R 20-25, 29, 31, 34-39, 41-43, 65, 66, 67	–
6. Chronic Toxicity	I/O	R 45-49, 60, 61, 64	R 33, 40, 62, 63	–
7. Endocrine Disruption Potential	I/O	R 50	R 51-58	no water hazard
8. Global Warming Potential	O	GWP > 20	GWP < 20	no global warming potential
9. Ozone Creation Potential	O	ODP > 0.5	ODP < 0.5	no ozone depetion potential
10. Acidification Potential	O	AP > 0.5	AP < 0.5	no acidification potential
11. Photochemical Ozone Creation	O	POCP > 30	2 < POCP < 30	POCP < 2
12. Odor	O	–	odor threshold < 300 mg m^{-3}	odor threshold > 300 mg m^{-3}
13. Eutrophication Potential	O	N-content > 0.2 or P-content > 0.05	N-content < 0.2 or P-content < 0.05	Compound without N and P
14. Organic Carbon Pollution Potential	O	–	ThOD > 0.2 g O_2 g^{-1} substrate	ThOD < 0.2 g O_2 g^{-1} substrate or no organic compound

Table S11. Equations used for calculation of weighting factors and indices.

Weighting factors/indices	Calculation
Environmental factor component *i*: EF_i [index points g^{-1}] via arithmetic average $EF_{Mw,i}$ and multiplication $EF_{Mult,i}$, where IGi,j is the value of component i in impact group *j* and *j* is the number of impact groups	$EF_{Mw,i}=\sum_{i=1}^{4} IG_{i,j}/j$<br>$EF_{Mult,i}=\prod_{i=1}^{4} IG_{i,j}$
Mass index component *i*: MIi [g g^{-1}] where m_i is the amount of component i [g] and m_p is the amount of final product [g]	$MI_i={}^{m_i}/_{m_p}$
Environmental index of the component i: EI_i [index points g^{-1}]	$EI_i=EF_i\cdot{}^{m_i}/_{m_p}=EF_i\cdot MI_i$
Environmental index of the process: calculated as EI_i or EI_{out} [index points g^{-1}]	$EI_{in}=\sum_{i=1}^{n_{in}} EI_i$<br>$EI_{out}=\sum_{i=1}^{n_{out}} EI_i$
General effect index of the process: GEI [index points g^{-1}]	$GEI={}^{EI_{process}}/_{MI_{process}}$

Table S12. The General Effect Index (GEI) calculation for Fe SACs production via self-assembly strategy[a]. g g^{-1}.

A, Input

Item	Mass index [g g^{-1}]	Impact factors							Environmental factor, [g^{-1}]		Environmental index, [g^{-1}]	
		1	2	3	4	5	6	7	EF_{Mw}	EF_{Mult}	EI_{Mw}	EI_{Mult}
melamine	3.0	C	B	C	A	B	C	C	0.4	6.76	1.20	20.28
cyanuric acid	2.4	C	C	C	C	B	C	C	0.075	1.3	0.18	3.12
Water	750	C	C	C	C	C	C	C	0	1.0	0	750
oxalic acid	0.45	C	C	C	C	B	C	C	0.075	1.3	0.034	0.585
Ar	0.48	C	C	C	C	C	C	C	0	1.0	0	0.48
ferric nitrate	0.48	C	B	C	C	B	C	C	0.15	1.69	0.072	0.8112
Total	756.81										1.486	775.28
GEI [g]											0.00196	1.02440

A, Output

Item	Mass index [g g^{-1}]	Impact factors											Environmental factor, [g^{-1}]		Environmental index, [g^{-1}]	
		4	5	6	7	8	9	10	11	12	13	14	EF_{Mw}	EF_{Mult}	EI_{Mw}	EI_{Mult}
Fe_1/CN	1.0	C	B	C	C	C	C	C	C	C	C	C	0.75	1.3	0.075	1.3
Losses	0.3	C	B	C	C	C	C	C	C	C	B	C	0.15	1.69	0.045	0.507
NH_3	5.03	B	B	C	C	C	C	C	C	B	C	C	0.225	2.197	1.132	11.051
Ar	0.48	C	C	C	C	C	C	C	C	C	C	C	0	1	0	0.48
Water	750	C	C	C	C	C	C	C	C	C	C	C	0	1	0	750
Total	756.81														1.252	763.34
GEI [g]															0.00165	1.00863

A, Input

Item	Mass index [g g^{-1}]	Impact factors							Environmental factor, [g^{-1}]		Environmental index, [g^{-1}]	
		1	2	3	4	5	6	7	EF_{Mw}	EF_{Mult}	EI_{Mw}	EI_{Mult}
melamine	3.0	C	B	C	A	B	C	C	0.4	6.76	1.20	20.28
cyanuric acid	2.4	C	C	C	C	B	C	C	0.075	1.3	0.18	3.12
Water	750	C	C	C	C	C	C	C	0	1.0	0	750
hydroxylamine hydrochloride	0.33	C	C	C	C	B	A	A	0.025	1.3	0.083	0.429
Ar	0.48	C	C	C	C	C	C	C	0	1.0	0	0.48
ferrous sulfate	0.33	C	B	C	C	B	C	B	0.15	1.69	0.050	0.5577
Total	756.54										1.512	774.87
GEI [g]											0.00200	1.02422

A, Output

Item	Mass index [g g^{-1}]	Impact factors											Environmental factor, [g^{-1}]		Environmental index, [g^{-1}]	
		4	5	6	7	8	9	10	11	12	13	14	EF_{Mw}	EF_{Mult}	EI_{Mw}	EI_{Mult}
Fe_1–Fe_1/CN	1.0	C	B	C	C	C	C	C	C	C	C	C	0.75	1.3	0.075	1.3
Losses	0.03	C	B	C	C	C	C	C	C	C	B	C	0.15	1.69	0.045	0.507
NH_3	5.03	B	B	C	C	C	C	C	C	B	C	C	0.225	2.197	1.132	11.051
Ar	0.48	C	C	C	C	C	C	C	C	C	C	C	0	1	0	0.48
Water	750	C	C	C	C	C	C	C	C	C	C	C	0	1	0	750
Total	756.54														1.211	762.88
GEI [g]															0.00160	1.00838

A, Input

Item	Mass index [g g^{-1}]	Impact factors							Environmental factor, [g^{-1}]		Environmental index, [g^{-1}]	
		1	2	3	4	5	6	7	EF_{Mw}	EF_{Mult}	EI_{Mw}	EI_{Mult}
melamine	3.0	C	B	C	A	B	C	C	0.4	6.76	1.20	20.28
cyanuric acid	2.4	C	C	C	C	B	C	C	0.075	1.3	0.18	3.12
Water	750	C	C	C	C	C	C	C	0	1.0	0	750
thioacetamide	0.45	C	C	C	C	B	A	B	0.075	1.3	0.09	0.468
Ar	0.48	C	C	C	C	C	C	C	0	1.0	0	0.48
ferrous sulfate	0.33	C	B	C	C	B	C	B	0.15	1.69	0.050	0.5577
Total	756.57										1.520	774.91
GEI [g]											0.00201	1.02423

A, Output

Item	Mass index [g g^{-1}]	Impact factors											Environmental factor, [g^{-1}]		Environmental index, [g^{-1}]	
		4	5	6	7	8	9	10	11	12	13	14	EF_{Mw}	EF_{Mult}	EI_{Mw}	EI_{Mult}
Fe_1–S–Fe_1/CN	1.0	C	B	C	C	C	C	C	C	C	C	C	0.75	1.3	0.075	1.3
Losses	0.06	C	B	C	C	C	C	C	C	C	B	C	0.15	1.69	0.009	0.101
NH_3	5.03	B	B	C	C	C	C	C	C	B	C	C	0.225	2.197	1.132	11.051
Ar	0.48	C	C	C	C	C	C	C	C	C	C	C	0	1	0	0.48
Water	750	C	C	C	C	C	C	C	C	C	C	C	0	1	0	750
Total	756.57														1.252	762.93
GEI [g]															0.00161	1.00841

[a] 1-14 refer to 14 environmental impact categories. A, B, and C are the degree of impact of each condition on each environmental category, and the relationship between the degree of influence is A > B > C. The classification criteria are detailed in Table 11. And A, B, and C in Table 12 correspond to red, organic, and green colors. The equations for calculating the input and output General Environmental Indices (GEI) values are detailed in Table 13, The smaller the value of the GEI computed for the input and output, the smaller the impact on the environment.

Table S13. Comparison of the GEI values of Fe_1/CN, Fe_1–Fe_1/CN, Fe_1–S–Fe_1/CN.

Catalysts	Input		Output	
	EI_{Mw}	EI_{Mult}	EI_{Mw}	EI_{Mult}
Fe_1/CN	0.00196	1.0244	0.00165	1.0086
Fe_1–Fe_1/CN	0.00200	1.3215	0.00160	1.0084
Fe_1–S–Fe_1/CN	0.00201	1.3215	0.00161	1.0084

Note: The GEI represents a weighted average of the Environmental Factors of all components involved, the lower the GEI value, the more friendly the process is to the environment.

Table S14. The HPLC analysis conditions for different substrates.

Substrates	Flow ($mL{\cdot}min^{-1}$)	λ (nm)	CH_3OH (%)	H_2O (%)	EA[a] (%)	EB[b] (%)	EC[c] (%)	Acetonitrile (%)
4-CP	1.0	225	55	45	–	–	–	50
p-nitrophenol	1.0	317	50	–	–	50	–	–
Phenol	1.0	280	40	60	–	–	–	–
Sulfamethoxazole	1.0	270	–	–	70	–	–	30
Tetracycline	1.0	357	–	–	–	20	–	80
Ciprofloxacin	1.0	278	–	–	–	60	–	40
Hydroxybenzoic acid /BQ	1.0	244	–	70	–	–	–	30
PMSO/$PMSO_2$	1.0	230	–	–	80	–	–	20

[a]: Eluent A, 0.1% CH_3COOH solution. [b]: Eluent B, 0.1% H_3PO_4 solution. [c]: Eluent C, 0.05% HCOOH solution

References

1. C. L. Chen, M. Z. Sun, F. Zhang, H. J. Li, M. R. Sun, P. Fang, T. L. Song, W. X. Chen, J. C. Dong, B. Rosen, P. W. Chen, B. L. Huang, Y. J. Li, *Energy Environ. Sci.* **2023**, *16*, 1685.
2. E. Vorobyeva, E. Fako, Z. P. Chen, S. M. Collins, D. C. Johnstone, P. A. Midgely, R. Hauert, O. V. Safonova, G. Vile, N. Lopéz, S. Mitchell, J. Pérez-Ramírez, *Angew. Chem. Int. Ed.* **2019**, *58*, 8724-8729.
3. Z. P. Chen, S. Mtichell, E. Vorobyeva, R. K. Leary, R. Hauert, T. Furnival, Q. M. Ramasse, J. M. Thomas, P. A. Midgley, D. Dontsova, M. Antonietti, S. Pogodin, N. López, J. Pérez-Ramírez, *Adv. Funct. Mater.* **2017**, *27*, 1605785.
4. Z. J. Li, Z. Y. Guo, X. Y. Wu, X. H. Jiang, H. Li, J. Xu, K. Yang, D. H. Lin, *ACS Nano* **2023**, *17*, 22859−22871.
5. Q. C. Xia, K. Jin, Y. L. Huang, Y. J. Zhai, W. K. Han, J. Wu, C. Xia, C. C. Lin, X. H. Zhao, X. Zhang. *Adv. Funct. Mater.* **2024**, *33*, 2314596.
6. X. Y. Wang, Y. J. Chen, F. Li, R. K. Miao, J. E. Huang, Z. L. Zhao, X. Y. Li, R. Dorakhan, S. L. Chu, J. H. Wu, S. X. Zheng, W. Y. Ni, D. H. Kim, S. J. Park, Y. X. Liang, A. Ozden, P. F. Qu, Y. Hou, D. Sinton, E. H. Sargent. *Nat. Commun.* **2024**, *15*, 616.
7. A. Jain, S. Y. Ong, G. Hautier, W. Chen, W. D. Richards, S. Dacek, S. Cholia, D. Gunter, D. Skinner, G. Ceder, K. A. Persson, *APL Mater.* **2013**, *1*, 011002.
8. P. H. Shao, J. Y. Tian, F. Yang, X. G. Duan, S. S. Gao, W. X. Shi, X. B. Luo, F. Y. Cui, S. J. Luo, S. B. Wang, *Adv. Funct. Mater.* **2018**, *28*,1705295.
9. M. Y. Xing, W. J. Xu, C. C. Dong, Y. C. Bai, J. B. Zeng, Y. Zhou, J. L. Zhang, Y. D. Yin, *Chem* **2018**, *4*, 1359−1372.
10. X. H. Jiang, Q. J. Xing, X. B. Luo, F. Li, J. P. Zou, S. S. Liu, X. Li, X. K. Wang, *Appl. Catal. B-Environ.* **2018**, *228*, 29–38.
11. F. Z. Ji, H. Zhang, X. X. Wei, Y. H. Zhang, B. Lai, *Chem. Eng. J.* **2019**, *359*, 1316−1326.
12. Z. Wang, J. Jiang, S. Y. Pang, Y. Zhou, C. T. Guan, Y. Gao, J. Li, Y. Yang, W. Qiu, C. C. Jiang, *Environ. Sci. Technol.* **2018**, *52*, 11276–11284.
13. J. J. Jiang, X. Y. Wang, Y. Liu, Y. H. Ma, T. R. Li, Y. H. Lin, T. F. Xie, S. S. Dong, *Appl. Catal. B-Environ.* **2020**, *278*, 119349.
14. J. K. Nørskov, J. Rossmeisl, A. Logadottir, L. Lindqvist, J. R. Kitchin, T. Bligaard, H. Jónsson, *J. Phys. Chem. B* **2004**, *108*, 17886–17892.
15. Z. W. Wang, W. L. Wang, J. Wang, Y. Yuan, Q. Y. Wu, H. Y. Hu, *Appl. Catal. B-Environ.* **2022**, *305*, 121049.

16. J. H. Cui, L. N. Li, Y. C. Wu, J. Y. Gao, K. Wang, C. Z. Diao, C. Hu, Y. B. Zhao, *Appl. Catal. B-Environ.* **2023**, *331*, 122706.
17. J. H. Cui, L. N. Li, S. T. Shao, J. Y. Gao, K. Wang, Z. C. Yang, S. Q. Zeng, C. Z. Diao, Y. B. Zhao, C. Hu, *ACS Catal.* **2022**, *22*, 14954−14963.
18. H. C. Zhang, P. X. Cui, D. H. Xie, Y. J. Wang, P. Wang, G. P. Sheng, *Adv. Sci.* **2023**, *10*, 2205681.
19. Y. Bao, C. Lian, K. Huang, H. R. Yu, W. Y. Liu, J. L. Zhang, M. Y. Xing, *Angew. Chem. Int. Ed.* **2022**, *61*, e202209542.
20. C. Cheng, W. Ren, F. Miao, X. T. Chen, X. X. Chen, H. Zhang, *Angew. Chem. Int. Ed.* **2023**, *62*, e202218510.
21. Y. Li, J. H. Hu, Y. B. Zou, L. Lin, H. B. Liang, H. X. Lei, B. Li, X. Y. Li, *Chem. Eng. J.* **2023**, *453*, 139890.
22. N. Jiang, H. D. Xu, L. H. Wang, J. Jiang, T. Zhang, *Environ. Sci. Technol.* **2020**, *54*, 14057–14065.
23. K. Qian, H. Chen, W. L. Li, Z. M. Ao, Y. N. Wu,, X. H. Guan, *Environ. Sci. Technol.* **2021**, *55*, 7034-7043.
24. B. K. Huang, Z. L. Wu, X. H. Wang, X. Y. Song, H. Y. Zhou, H. Zhang, P. Zhou, W. Liu, Z. K. Xiong, B. Lai, *Environ. Sci. Technol.* **2023**, *57*, 15667−15679.
25. Z. D. Zhao, M. Z. Hu, T. T. Nie, W. J. Zhou, B. C. Pan, B. S. Xing, L. Z. Zhu, *Environ. Sci. Technol.* **2023**, *57*, 4556−4567.
26. M. Q. Li, H. Li, C. C. Ling, H. Shang, H. Wang, S. X. Zhao, C. Liang, C. L. Mao, F. R. Guo, B. Zhou, Z. H. Ai, L. Z. Zhang, *Proc. Natl. Acad. Sci.* **2023**, *120*, e2304562120.
27. C. Liang, H. W. Sun, C. C. Ling, X. F. Liu, M. Q. Li, X. Zhang, F. R. Guo, X. Zhang, Y. B. Shi, S. Y. Cao, H. He, Z. H. Ai, L. Z. Zhang, *Water Res.* **2023**, *228*, 119328.
28. Y. F. Lin, Y. Wang, Z. L. Weng, Y. Zhou, S. Q. Liu, X. W. Ou, X. Xu, Y. P. Cai, J. Jiang, B. Han, Z. F. Yang, *Nat. Commun.* **2024**, *15*, 10032.
